# DeltaDTM: A global coastal digital terrain model

**DOI:** 10.1038/s41597-024-03091-9

**Published:** 2024-03-06

**Authors:** Maarten Pronk, Aljosja Hooijer, Dirk Eilander, Arjen Haag, Tjalling de Jong, Michalis Vousdoukas, Ronald Vernimmen, Hugo Ledoux, Marieke Eleveld

**Affiliations:** 1 Deltares, Delft, Netherlands; 2 Delft University of Technology, Delft, Netherlands; 3 University of the Aegean, Mitilini, Greece; 4 Data for Sustainability, Axel, Netherlands

**Keywords:** Geography, Geomorphology

## Abstract

Coastal elevation data are essential for a wide variety of applications, such as coastal management, flood modelling, and adaptation planning. Low-lying coastal areas (found below 10 m +Mean Sea Level (MSL)) are at risk of future extreme water levels, subsidence and changing extreme weather patterns. However, current freely available elevation datasets are not sufficiently accurate to model these risks. We present DeltaDTM, a global coastal Digital Terrain Model (DTM) available in the public domain, with a horizontal spatial resolution of 1 arcsecond (∼30 m) and a vertical mean absolute error (MAE) of 0.45 m overall. DeltaDTM corrects CopernicusDEM with spaceborne lidar from the ICESat-2 and GEDI missions. Specifically, we correct the elevation bias in CopernicusDEM, apply filters to remove non-terrain cells, and fill the gaps using interpolation. Notably, our classification approach produces more accurate results than regression methods recently used by others to correct DEMs, that achieve an overall MAE of 0.72 m at best. We conclude that DeltaDTM will be a valuable resource for coastal flood impact modelling and other applications.

## Background & Summary

With the Space Radar Topography Mission (SRTM) and the introduction of its synonymous named dataset–the first global digital elevation model (DEM) with a resolution of 3 arcseconds–by NASA in 2004^1^, new global applications of elevation data became possible. These include, among others, watershed modelling, slope impact, and surface water modelling^2^.

Subsequent missions and their datasets, such as ALOS, ASTER and TanDEM-X have improved on vertical accuracy and horizontal spatial resolution, reaching 1 arcsecond (roughly 30 m at the equator). While higher resolution datasets are commercially available, the most vertically accurate^3^ freely available global high-resolution DEM is CopernicusDEM^4^ with a resolution of 1 arcsecond, based on the TanDEM-X mission data.

However, these missions, using X-band radar (SRTM, TanDEM-X) and optical sensors in the visible spectrum (ALOS, ASTER), measure the upper part of canopy and buildings. That makes their datasets Digital Surface Models (DSM) rather than Digital Terrain Models (DTM), as they do not represent the bare-earth everywhere. The differences between the surface and terrain can be tens of meters for vegetated areas. While some applications can use these DSMs, accurate flood (impact) modelling requires DTM data^5^. Indeed, issues such as future extreme water levels due to Sea Level Rise (SLR)^6,7^, subsidence and the worsening of storm surges, require *terrain* elevation data having higher accuracy (within 1 m), and for all the coastal areas of the world. For this purpose, local airborne lidar data is sometimes used, but this is expensive and not available globally, only in more affluent parts of the world. This discrepancy has been noted by several authors^5,8,9^, but many flood studies nonetheless use DSMs as input.

Attempts have been made to correct biases for areas covered by vegetation or buildings in global DEMs–thus approximating a DTM–by relying on auxiliary datasets such as tree-cover or urban agglomeration maps. Examples are MERIT^10^, based on SRTM, and CoastalDEM^11^, based on NASADEM^12^, itself the latest iteration of SRTM. More recently FABDEM^13^ and DiluviumDEM^14^–both based on CopernicusDEM–have been released. We denote these global DTMs as *corrected-DSMs*, and an overview is given in Table 1.

**Table 1:** Overview of corrected-DSMs.

Name	Year	Based on	Auxiliary input	Resolution	Correction	Licence
MERIT^10^	2017	SRTM	ICESat-1, Tree density, Tree height	3”	Regression techniques	CC-BY-NC 4.0/Open Database licence
CoastalDEM^11^	2020	NASADEM	ICESat-2	1”	Neural network	Commercial/free for research only
FABDEM^13^	2022	CopernicusDEM	Canopy height, WorldPop, World Settlement Footprint among others	1”	Two decision tree models	Commercial/free for research only
DiluviumDEM^14^	2023	CopernicusDEM	Canopy height, Landsat Cloud Cover, Dynamic World among others	1”	Gradient boosted decision tree	CC-BY 4.0
DeltaDTM (this study)^17^	2023	CopernicusDEM	ICESat-2^25^, GEDI^26^, ESA WorldCover^27^	1”	Morphological filters, spatial interpolation	CC-BY 4.0

ICESat (2003–2010) was the first spaceborne lidar mission which enabled bare-earth elevation measurements globally, even in forests, but was very limited in the amount of data it could collect. However, since 2018, the spaceborne lidar missions ICESat-2 and GEDI enable global terrain measurements on a much larger scale. The properties of these missions are given in Table 2. ICESat-2 has been used on its own to create a global coastal digital terrain model (GLL_DTM v2^15^), which results in high accuracy (MAE of 0.34 m) but low horizontal spatial resolution of ~1 km.

**Table 2:** Characteristics of the ICESat-2 and GEDI space borne lidar missions.

Mission	ICESat-2	GEDI
Type	Discrete photon	Full waveform
Main objective	Cryosphere monitoring	Global ecosystems
Duration	2018–2024 (ongoing)	2019–2023
Orbit Inclination	92	51.6
Beam footprint	11 m	23 m
# tracks	6 (in 3 strong/weak pairs)	8 (four strong, four weak)
Along track spacing	0.7 m (20 m for ATL08)	70 m
Across track spacing	3 km/90 m between pair	0.6 km
Swath width	6.6 km	4.2 km
Beam frequency	532 nm (green)	1064 nm (near-infrared)
Vertical accuracy	0.91 cm MAE^32^	1.80 cm MAE^32^

For achieving higher resolution DEMs with spaceborne lidar, such data must be combined with global DEMs. As Magruder *et al*.^16^ suggests, both CoastalDEM^11^ and more recently FABDEM^13^ and DiluviumDEM^14^, use ICESat-2 data to correct the surface data present in global DEMs. However these corrected-DSMs, with the exception of DiluviumDEM, are not in the public domain–they are only free for research purposes–nor are the machine learning models used to generate them (see Table 1).

We introduce DeltaDTM^17^, a fully open and reproducible global coastal DTM with 1 arcsecond resolution, based on CopernicusDEM (and thus also a corrected-DSM), ICESat-2 and GEDI data. Here, coastal is defined as the Low Elevation Coastal Zone (LECZ) below 10 m + MSL^18^, which is the area most affected by future extreme water levels^8^ and storm surges^19^. When DeltaDTM is compared with airborne lidar validation data across the world, the mean absolute error (MAE) across all land cover classes is 0.45 m with 91% of cells accurate within 1 m, compared to 0.72 m and 79% for the most accurate other high resolution product (the recently released DiluviumDEM). We conclude that DeltaDTM will be a valuable resource for coastal flood impact modelling and other applications.

## Methods

DeltaDTM is a global coastal DTM based on a fusion of CopernicusDEM, ICESat-2, and GEDI elevation data. We remove the vertical biases of surface data (e.g., canopy, buildings) present in CopernicusDEM by using ICESat-2 and GEDI *terrain* elevation measurements. Our method can be broken down into four categories, as discerned by Okolie *et al*.^20^ in their review of DEM fusion methods.

- *Spatial filtering*, such as removing pits present in CopernicusDEM (similar to FABDEM^13^) and other outliers.
- *Co-registration*, to vertically align CopernicusDEM and ICESat-2, thereby removing the vertical bias in CopernicusDEM (similar to NASADEM^12^ using ICESat)
- *Filtering of non-ground points*, by classifying CopernicusDEM into terrain and non-terrain using morphological filters^21^ and removing the non-terrain elevation pixels.
- *Void filling*, by spatially interpolating the values removed in the previous step, using the AIDW method^22^.

**Fig. 1:**
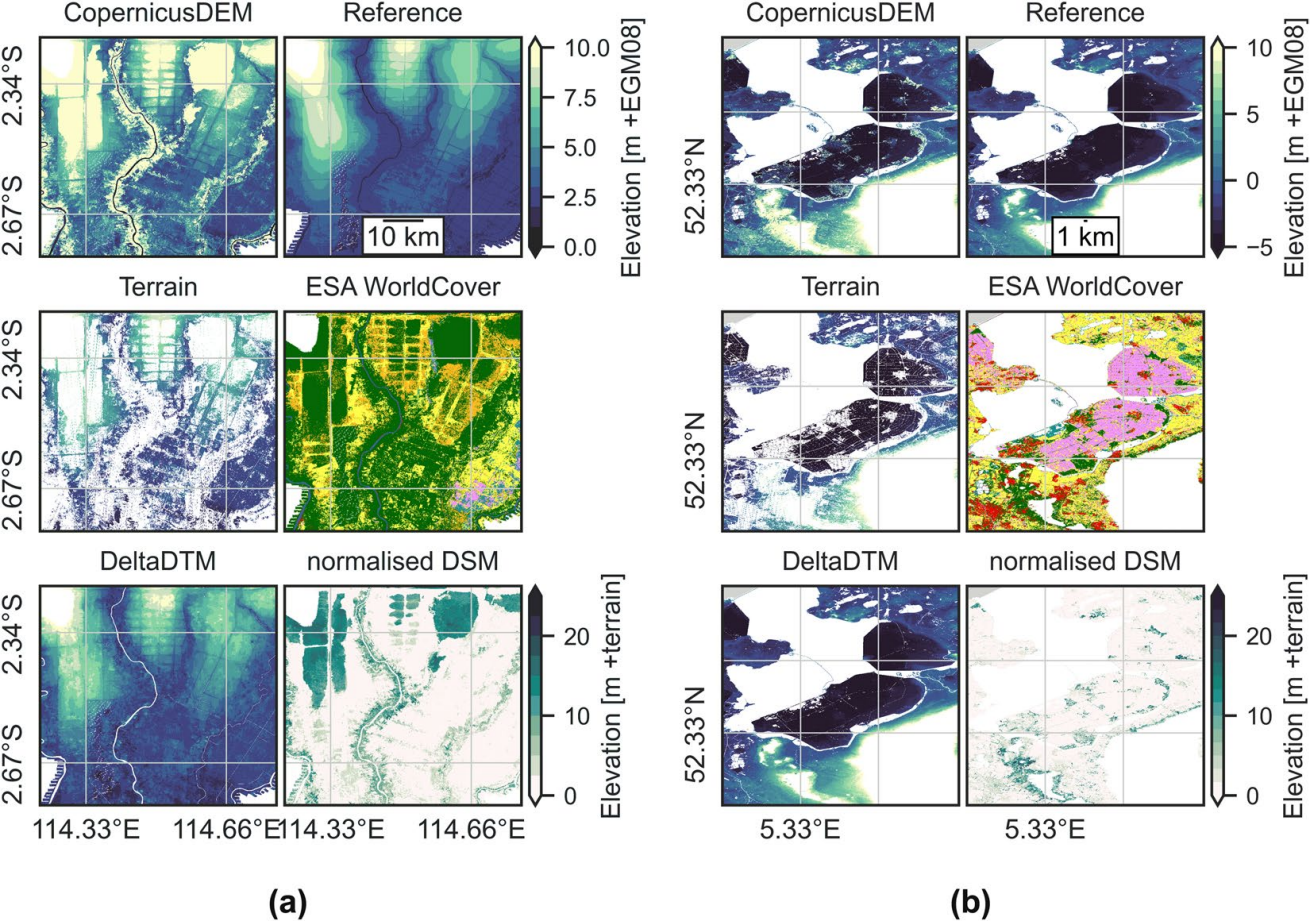
Explanation of the classification process of DeltaDTM in (**a**) Kalimantan and (**b**) the Netherlands. The top row shows *CopernicusDEM*–the input DSM for DeltaDTM–and the *Reference* airborne lidar DTM for this area. The middle row shows the classification of the *Terrain* pixels, with the *ESA WorldCover* map as reference. The bottom row shows *DeltaDTM*, the result of the interpolation of *Terrain*, with the *Normalised DSM* as reference. The normalised DSM is created by subtracting DeltaDTM from CopernicusDEM, resulting in a map of surface heights above the terrain.

**Fig. 2:**
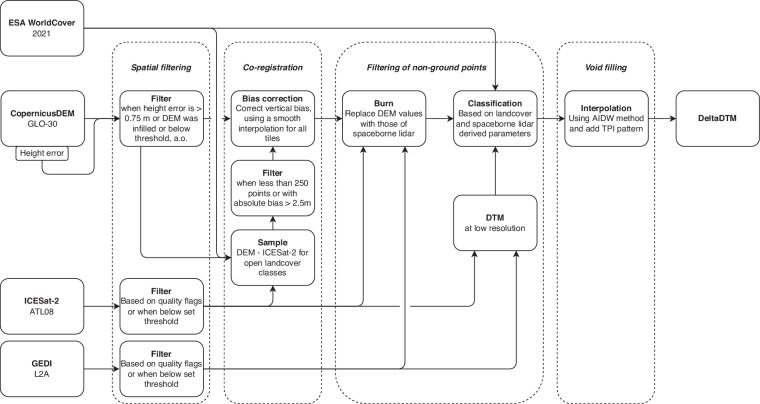
Overview of the DeltaDTM workflow. Each box is a dataset or processing step, with the dotted line grouping all the preprocessing steps.

A visual explanation of the last two steps–filtering and interpolation–is given in Fig. 1. A complete overview of the approach is given in Fig. 2, where each box is a dataset or processing step. Each of these steps is explained in detail below.

We differ from other corrected-DSMs (e.g., FABDEM) in the use of classification–instead of regression–in order to find the terrain height. While this leads to a theoretical loss of resolution (when data is filtered and afterwards interpolated), we find this drawback is negated by the improved accuracy, especially in data-scarce areas.

### Datasets used

The datasets used to create DeltaDTM are listed in Table 3. All datasets are publicly available and only require citing for use.

**Table 3:** Input datasets used.

Dataset	Measures	Type	Years collected	Based on	Resolution	Size
CopernicusDEM^4^	Elevation	raster	2012–2015	TanDEM-X	1” or 30 m	500 GB
ICESat-2 ATL08^24,25^	Elevation	lidar points	2018–2023	ICESat-2 ATL03	20 m along-track	22200 GB
GEDI L2A^26^	Elevation	lidar points	2018–2023	—	70 m along-track	107200 GB
ESA-WorldCover^27^	Land cover	raster	2021	Sentinel-1, Sentinel-2	10 m	117 GB

The spaceborne lidar datasets contain much more (meta)data than latitude, longitude, and height, which makes them much larger than their raster counterparts. These sizes represent the full global coverage of these datasets, as there is no possibility to only download a subset for the global low elevation coastal zone (LECZ).

The base elevation model we use as starting point is the CopernicusDEM GLO-30 dataset, provided under COPERNICUS by the European Union and ESA^4^. The dataset is distributed in tiles of 1 degree by 1 degree, with a spatial resolution of one arcsecond (~30 m at the equator). It is based on TanDEM-X interferometric synthetic aperture radar (SAR) data^23^ and is freely available for the entire globe, except Armenia and Azerbaijan, due to current export restrictions. Each elevation tile is accompanied by both a water mask and height error tiles, which we also use in our analysis.

For vertically more accurate–but sparsely distributed–terrain elevation measurements, we use the ICESat-2 Level 3 Land and Vegetation height (ATL08) product^24,25^, at version 6, with dates ranging from 2018-10-14 to 2023-06-22. We downloaded 262807 granules (totalling ~22 TB) from the NSIDC DAAC. For elevation, we use the *h_te_best_fit_20 m* (best fit of all terrain photons in a 20m segment) field, containing the elevation above the WGS84 ellipsoid and related latitude *latitude_20m* and longitude *longitude_20m* fields for each track group in the HDF5 file.

Similarly, we downloaded 74815 granules (totalling ~107 TB) of the Global Ecosystem Dynamics Investigation (GEDI) Level 2 A product^26^, currently at version 2, with dates ranging from 2019-04-18 to 2023-03-16. We use the *elev_lowestmode* field, containing the terrain elevation above the WGS84 ellipsoid and related latitude *lat_lowestmode* and longitude *lon_lowestmode* fields for each track group in the HDF5 file.

We also sample the land cover class from the ESA WorldCover 2021 dataset^27^ for further use in both the bias correction and classification algorithms. This land cover class dataset is chosen because it is recent and has a resolution of ~10 m, which exceeds CopernicusDEM. The data is freely distributed in tiles of 3 degree by 3 degree, which we resample (by majority) to the tile specification of CopernicusDEM. WorldCover recognizes several land cover classes, such as “Grassland”, “Cropland”, “Tree cover” and “Built-up”. Specifically for our bias correction and filtering, we denote the classes “Shrubland”, “Grassland”, “Cropland”, “Bare”, “Moss” and “Snow” as *open* land cover (i.e. terrain that is not covered by woody vegetation or buildings), and the remaining classes “Tree cover”, “Mangroves”, and “Built-up” as *closed* land cover. We assume elevation values in CopernicusDEM for open land cover to approximate terrain measurements, whereas elevation values in *closed* land cover do not.

#### Preprocessing

We find all CopernicusDEM tiles that contain values below 10 m + MSL by intersection with the GLL_DTM^15^ and a manual inspection of elevation along floodplains. This results in a subset of 7146 tiles, out of a total of 26448.

We tile all datasets to the tile specification of the CopernicusDEM tiles in this subset. This streamlines processing and enables parallel processing of tiles. To prevent edge artefacts on tile borders, each tile is processed with 5% overlap of the neighbouring tiles. This overlap percentage is a safety margin as subsequent filters and void filling could require a 12 km buffer.

### Spatial filtering

CopernicusDEM, ICESat-2, and GEDI datasets all contain outliers. Whereas outliers with elevations greater than the actual terrain will be automatically removed in the subsequent classification step (as if they are buildings or canopy), outliers with elevations lower than the actual terrain are problematic as they will be classified as terrain as well and negatively influence the classification of surrounding cells. Here we describe the outlier filters for each dataset, with the focus on removing low outliers.

#### CopernicusDEM

CopernicusDEM contains many small low outliers, often the result of multi-bounce backscattering errors in urban areas, such as around electricity poles. We apply a 25 by 25 pixel window (~750 × 750 m) function, and remove all values below 2 standard deviations of all elevation values in the window. The window size is sufficient to filter larger patches (3 by 3 pixels) of low outliers, as observed in CopernicusDEM. Furthermore, for each 1 by 1 tile we determine an elevation cut-off value, below which all data is removed. By default it is set to −2 m, with manual corrections for lowest-lying areas, such as polders in the Netherlands, which has been set to −7 m. This value is provided as the *low_cutoff* field in the *tiles.gpkg* geospatial database supplied with the DEM. Resulting gaps are filled by void filling as described in subsequent steps, using overlapping tiles to prevent any edge artefacts.

Likewise, based on the height error data provided with CopernicusDEM^4,28^, we remove all elevation values for which the height errors exceed 0.75 m or the DEM was infilled with another DEM. These values are empirically chosen based on the outliers observed in validation areas. Around 1% of values in CopernicusDEM are removed this way. We note that FABDEM^13^ similarly attempts to remove low outliers in CopernicusDEM, but does so by repeatedly smoothing the elevation values.

#### Spaceborne lidar

We apply quality filters on both the ICESat-2 and GEDI data. For ICESat-2 we only keep data with the flag *subset_te_flag* set to 1. For GEDI, we apply the filtering as used for the derived GEDI L3A product^29^ and keep only data with the *sensitivity* flag above 0.95. Like the filter used for CopernicusDEM, we also remove all values below 2 standard deviations of all elevation values in a ~750 × 750 m window. We also use the same elevation cut-off value, by default set to −2 m, below which all data is removed. These filters remove around 1% of measurements.

Both ICESat-2 and GEDI elevation values were vertically transformed using PROJ^30^ from the ellipsoid to the Earth Gravitational Model (EGM2008) geoid^31^ (assumed to approximate global MSL in this study), the same vertical reference as used by CopernicusDEM.

### Co-registration

Any elevation dataset will have biases due to instrument and processing errors, and these biases can be determined and corrected by using a second–more accurate–elevation dataset. Indeed, the first ICESat mission has been used to validate the CopernicusDEM dataset^28^, and Guth *et al*.^3^ used ICESat-2 to validate several global DEMs. We use the ICESat-2 ATL08 data to correct the terrain elevation bias in the CopernicusDEM dataset. GEDI is not used for the bias correction, as it is less accurate for terrain elevation assessment than ICESat-2^32^ and does not cover latitudes above 56. The bias correction is the first step in the workflow, so the terrain elevation values of ICESat-2 and CopernicusDEM are aligned in subsequent steps.

**Fig. 3:**
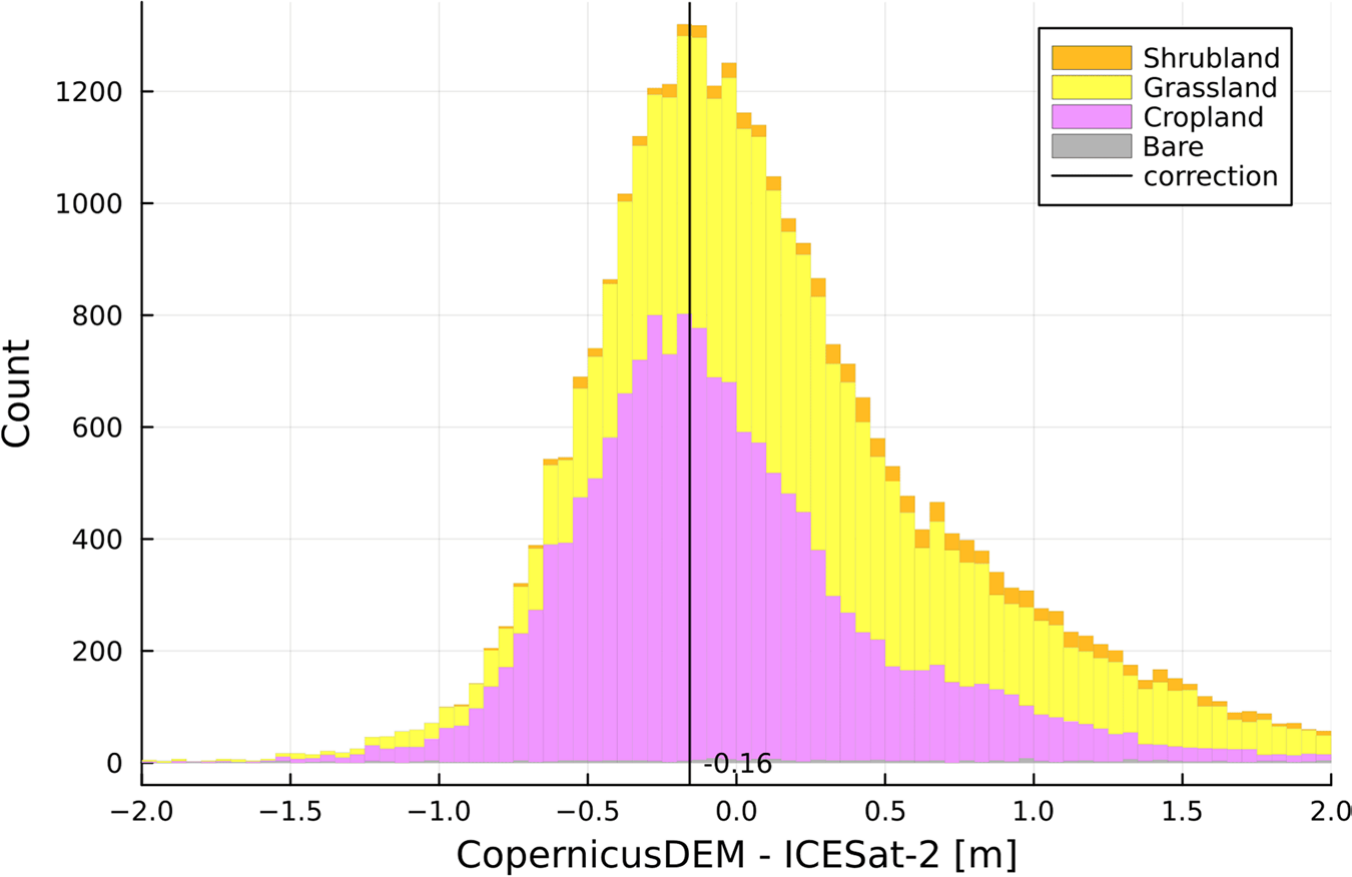
A (stacked) distribution of the difference between CopernicusDEM and ICESat-2 ATL08 elevation values for all open WorldCover land cover classes for 1/4 th of the S04-E114 CopernicusDEM tile. Here, based on the most measured (peak) differences (vertical correction line in black), we determine this part of the CopernicusDEM tile to be 16 cm lower than ICESat-2. The different individual open land cover classifications–colour coded like WorldCover–are given as a reference, but are not used in the calculation.

**Fig. 4:**
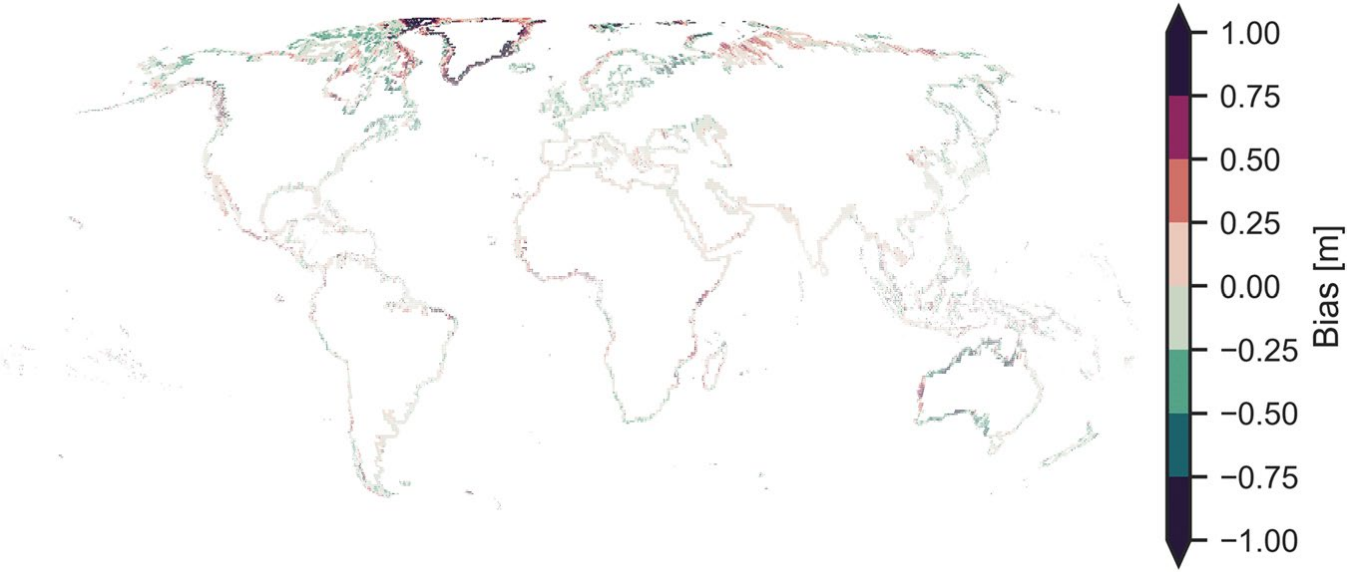
Global mean error (bias) of CopernicusDEM for each 1/4 th tile when compared with ICESat-2 elevations for open land covers.

For each quarter of a CopernicusDEM tile (0.5 by 0.5), we compare (subtract) the elevation of the ICESat-2 points to the elevation of the CopernicusDEM data for all open land covers from WorldCover. This is the smallest subdivision of a tile which consistently yields hundreds of ICESat-2 measurements, so the bias can be estimated with certainty. In this way, the distribution of CopernicusDEM minus ICESat-2 could be calculated and the peak of this distribution was denoted as the bias for each tile. An example for a single CopernicusDEM tile is given in Fig. 3, where a bias of −0.16 m was found. The values of the biases for all tiles are given in Fig. 4.

We excluded tiles with a bias larger than 2.5 m or those with less than 250 data points from the bias correction. These parameters were found empirically to remove gross outliers, mostly present at the poles and tiles containing small rocky islands. Large biases indicate a non-random error in CopernicusDEM (such as interaction with snow and ice), while a small number of points cannot be representative for a larger area, given that we can expect thousands of ICESat-2 points in a quarter of a tile. Overall these filters removed less than 1% of the tiles and kept the major coastlines intact (Fig. 4).

The resulting point dataset, containing the bias at the centre coordinates of each quarter of a CopernicusDEM tile, was used to create a bias correction raster for the whole tile by interpolating using a nearest neighbour algorithm. Afterwards, this bias correction raster was applied to the original CopernicusDEM tile. Overall we found an average bias of −0.03 m for the coastal tiles.

### Filtering of non-ground points

CopernicusDEM–like any current global radar or optical based DEM–measures the surface of the earth and thus includes vegetation, building heights, and other civil constructions. To remove these biases and determine the true “bare earth” surface, we apply morphological surface filters which are supported by terrain measurements from the ICESat-2 ATL08 and GEDI L2A data. Morphological filters relate to the morphology (shape) of features and work on subsections (windows) of raster (image) data, to which non-linear (such as minimum) filters are applied^33^. These filters are often used for terrain classification of airborne lidar datasets, like the Progressive Morphological Filter (PMF) by Zhang *et al*.^21^ or the Simple Morphological Filter (SMF) by Pingel^34^, but require at least some terrain measurements in a given area to work. On its own, CopernicusDEM is not suitable for such filtering, as it does not contain any terrain measurements in large parts of the world, such as tropical forests. Moreover, these filters normally operate on the scales of individual trees and houses, using raster resolutions of a metre, not ~30 m in the case of CopernicusDEM.

We replace CopernicusDEM data with ICESat-2 ATL08 and GEDI L2A terrain data when available, “burning” the lidar derived elevations into the bias-corrected CopernicusDEM raster. On average, this replaces 4% of the values in a tile. This enables the use of morphological filters, albeit with much larger windows sizes than usual morphological filters operations.

**Fig. 5:**
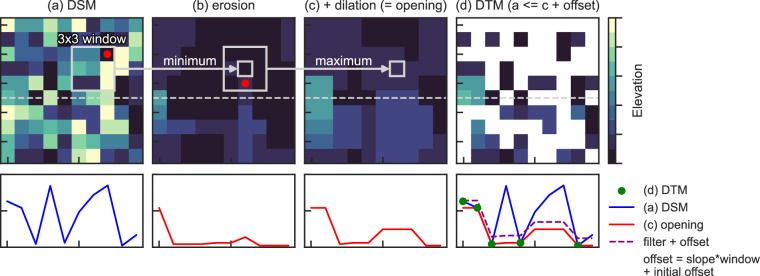
A diagram explaining the progressive morphological filter for classifying terrain and non-terrain values. The top panels are elevation rasters, with the bottom panels cross-sections (indicated by the grey dotted horizontal lines) of the top panel. A *morphological* opening operation (erosion in (**b**) followed by dilation in (**c**)) using a three by three window is applied on (**a**). A red dot is given for the location of the minimum and maximum value in each window. The resulting surface, plus an offset determined by the window size is the threshold surface for the binary terrain/non-terrain classification: All values in (**a**) below or equal to (c + offset) are classified as terrain. The offset is determined by the slope times the window size and an initial offset, where both the slope as the initial offset depend on the landscape and are set by the user. The filter is *progressive* by repeating the operation for increasingly larger window sizes, taking the minimum of all surfaces for use in the final classification.

We modify the PMF filter of Zhang *et al*.^21^ (explained in Fig. 5), by allowing for filter settings per raster cell instead of single static parameter and by allowing only erosion instead of the default opening operation. Thus, specific algorithm settings–such as slopes and the initial height threshold–are dynamically derived from ICESat-2 ATL08 and GEDI L2A data (Table 4).

**Table 4:** Settings of the classification algorithms in use.

Algorithm	PMF (erosion)	PMF (erosion)
Applied to	Open land cover	Closed land cover
Radius	1000 m	2000m
Slope	dynamic with a minimum of $1.0\,\frac{{\rm{m}}}{{\rm{km}}}$	dynamic with a minimum $1.0\,\frac{{\rm{m}}}{{\rm{km}}}$
Initial threshold	dynamic with a minimum of 1.0 m	dynamic with a minimum of 1.0 m

In particular, we create a low-resolution (~450 m) DTM from ICESat-2 and GEDI data during processing, and derive the slopes and the initial height settings for the morphological filter from it. This is the highest resolution DTM currently attainable without needing to interpolate more than 20 of the grid cells between the sparse ICESat-2 and GEDI points. The low-resolution DTM is similar to GLL_DTM v2^15^, but it includes GEDI data and includes elevations above the 10 m threshold to accurately represents slopes at the edge of the LECZ. This DTM–after bilinear resampling to ~30 m–is also used to detrend (by subtracting it from) the burned CopernicusDEM data, after which the PMF filter is run on it.

The slope derived from a low-resolution DTM can underestimate the slope of the terrain when applied at 30 m, as features smaller than the resolution of the DTM are not captured. For closed WorldCover land cover classes–such as “Tree cover” where the CopernicusDEM surface does not describe the terrain and requires filtering–we calculate the slope from the ~450 m lidar DTM as is. For open land cover classes–where little to no data is expected to be removed–we are less strict and use a slope value as if it were retrieved from a 30 m resolution DTM by dividing the grid size for which the slope is calculated by fifteen ($\frac{450{\rm{m}}}{30{\rm{m}}}$). We thus allow for steeper terrain features in open land cover classes than in closed land cover classes. The remainder of the settings are chosen based on experiments with validation data and are provided in Table 4.

### Void filling

**Fig. 6:**
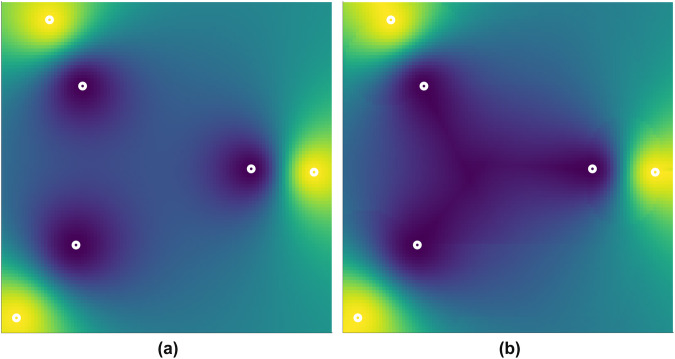
A diagram comparing (**a**) the IDW interpolation and (**b**) the interpolation with the AIDW method. A complete surface is interpolated from the six points in white, where the inner three points share a value, which differs from the value shared by the outer three points. This pattern will lead to the *bulls eye* artefact (concentric areas) in the IDW in (**a**), whereas the AIDW method in (**b**) adjusts the weighing of the outer points in the centre area to prevent this.

The resulting non-terrain cells–on average 50% of a tile–are filled by interpolation using the Adjusted Inverse Distance Weighing (AIDW) method by Li^22^ using the remaining terrain points. This method is a standard IDW method, but with lower weights for points that are “behind” closerby points, in respect to the interpolated point. In effect, this ensures that the values used are from all around a point, instead of just close-by points in one direction. This property prevents the use of elevation values from only a single ICESat-2 or GEDI track. An example highlighting the differences between IDW and AIDW is given in Fig. 6.

The resulting interpolated surface is unrealistically smooth for a terrain. To create a more realistic visual landscape representation, we add the roughness of the surface–derived from the original CopernicusDEM–to the interpolated terrain values only. The roughness or Topographic Position Index (TPI)^35^ is the difference between the elevation of a pixel and the mean elevation of its eight neighbours. As these values can sometimes be several metres, we limit (clamp) them to range of the initial threshold (used in the PMF filter) derived from the low-resolution DTM. So, in case the initial threshold is 1.2 m, there is a range of −0.60.6 m, and all TPI values above 0.6 m will be set to 0.6 m, and all values below −0.6 m to −0.6 m. In the worst case, this adds random noise to the DEM, like the noise present in non-interpolated CopernicusDEM elevation values. However, in the best case, it represents actual topography patterns, such as ditches or small canals underneath the canopy. Overall, the additions are small and balanced (roughly have a zero mean) and do not affect the accuracy.

## Data Records

DeltaDTM^17^ is available as a zipped (.zip) archive per continent (for a total of 20 GB) at 10.4121/21997565. It contains 7073 tiles of 1 by 1 degree with a spatial resolution of one arcsecond (~30 m) in the Cloud Optimized Geotiff (COG) format (.tif), using ZSTD compression. The names of the tiles are constructed by using the name DeltaDTM, a version v1_0 specifier, and the tile location NYYEXXX, split by underscores _. The tile location represents the coordinate of the top left corner of the tile, with N/S (North or South) and E/W (East or West) indicating the hemispheres, and the YY and XXX representing the latitude and longitude of the tile in that hemisphere, respectively. This location naming scheme is identical to the CopernicusDEM dataset. An example filename DeltaDTM_v1_0_S01W161.tif thus represents a DeltaDTM version 1.0 tile with the top left corner at 1 South (−1 latitude) and 161 West (−161 longitude).

The coordinate reference system (CRS) of the geotiffs is set to EPSG:9518, a compound CRS combining the horizontal geographic CRS WGS84 (EPSG:4326) reference and the vertical CRS EGM2008 (EPSG:3855). All geotiffs also contain metadata describing the dataset, usage notes and a reference to the doi of the dataset.

For each elevation tile, a mask tile is also provided in the mask_tiles.zip. These are the original CopernicusDEM water body mask tiles, with the addition of value 255 to indicate where DeltaDTM is clipped at 10 m + MSL.

A Virtual Raster Table (DeltaDTM_v1_0.vrt) file linking all tiles is also provided and is advised to be used for overviews or visualisation. Note that the Virtual Raster Table file cannot represent all the different sizes–becoming smaller at higher latitudes–of the individual tiles (also see the usage notes).

Finally, a geopackage (deltadtm_tiles.gpkg) is provided with the bounding boxes of each tile, including an attribute detailing in which zipfile (continent) it can be found. w The DeltaDTM dataset is publicy hosted as a Google Earth Engine collection under the collection ID *users/maartenpronk/deltadtm/v1*. An example on how to access the dataset is provided at https://code.earthengine.google.com/?scriptPath=users/maartenpronk/deltadtm:v1.

## Technical Validation

We validate DeltaDTM, global high-resolution DSMs (NASADEM, CopernicusDEM) and corrected-DSMs (MERIT, CoastalDEM, FABDEM, and DiluviumDEM) against local DTMs based on airborne lidar, and also inspect the differences visually. Furthermore, we globally cross-validate DeltaDTM against ICESat-2, demonstrating that DeltaDTM is currently the most accurate global coastal DTM.

### Validation against reference datasets

**Fig. 7:**
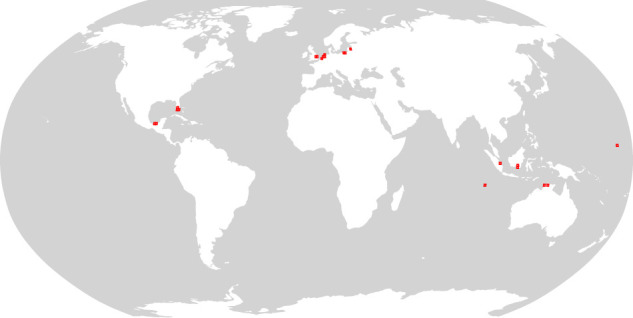
The validation areas from Table 5 projected on the globe.

We validate the DeltaDTM dataset against public local airborne lidar reference datasets in the Australia, Florida, Indonesia, Latvia, the Marshall Islands, Mexico, the Netherlands, Poland, and the United Kingdom. These datasets–with a combined area of 78106 km^2^–cover coastal areas across the world near or below MSL. An overview of the areas and the source data is given in Table 5 and Fig. 7. All datasets were vertically reprojected to MSL (using the EGM2008 geoid) from their local vertical references using GDAL^36^. We use the following metrics to evaluate the quality of the DeltaDTM dataset:

1 $${\rm{Mean}}\;{\rm{Error}}\;{\rm{(bias)}}=\frac{1}{n}\mathop{\sum }\limits_{i=1}^{n}\left({z}_{i}-{c}_{i}\right)$$

2 $${\rm{Mean}}\,{\rm{Absolute}}\,{\rm{Error}}\,{\rm{(MAE)}}=\frac{1}{n}\mathop{\sum }\limits_{i=1}^{n}\left|{z}_{i}-{c}_{i}\right|$$

3 $${\rm{Median}}\;{\rm{Absolute}}\;{\rm{Deviation}}\;{\rm{(MAD)}}=median\left(\left|({z}_{i}-{c}_{i})-median({z}_{i}-{c}_{i})\right|\right)$$

4 $${\rm{Root}}\,{\rm{Mean}}\,{\rm{Square}}\,{\rm{Error}}\,{\rm{(RMSE)}}=\sqrt{\frac{1}{n}\mathop{\sum }\limits_{i=1}^{n}{\left({z}_{i}-{c}_{i}\right)}^{2}}$$

Where *z*_*i*_ is the elevation of one cell in DeltaDTM and *c*_*i*_ the elevation in the airborne lidar reference dataset.

**Table 5:** Reference lidar datasets used.

Country	Dataset	Resolution	Year	Area [km^2^]	Most Common Land covers [%]
Australia	National DTM, near Darwin^43^	5 m	2001–2015	4137	Tree [41], Grassland [30]
Florida	NOAA Sea Level Rise^44^	2 m	2012	20754	Tree [28], Built-up [19]
Indonesia	East Sumatra strip DTM^45^	25 m	2014–2017	1499	Tree [49], Grassland [18]
Indonesia	Central Kalimantan^45^	25 m	2011	6529	Tree [60], Grassland [21]
Latvia	National DTM, near Riga^46^	20 m	2013–2019	2385	Tree [57], Grassland [22]
Marshall Islands	Majuro^47^	1 m	2017	581	Tree [37], Grassland [34]
Mexico	National DTM, Tabasco^48^	5 m	2011	13942	Wetland [37], Grassland [36]
the Netherlands	AHN4^49^	5 m	2020–2022	22685	Grassland [47], Cropland [25]
Poland	National DTM, near Gdańsk^50^	5 m	2021	1873	Cropland [70], Grassland [15]
United Kingdom	National DTM, Fenlands^51^	10 m	2019	3721	Cropland [69], Grassland [20]

Trees is “Tree cover”, while Wetland is “Herbaceous wetland”. Area is the area below 10 m + MSL.

The mean error (or bias) and RMSE metrics are commonly used, but are sensitive to outliers. We thus also provide the MAE and MAD metrics, which are more robust to outliers and are–in our opinion–the metrics that matter most. Furthermore, we also provide the number of sampled cells, and the percentage of values within 1, 2, and 5 m from the airborne lidar reference surface.

We calculate these metrics per land cover class as sourced from ESA WorldCover and provide an overall (all land cover classes combined) height error statistic (Table 6). Land cover classes that occur in less than 1% of all samples (“Snow and Ice”, “Herbaceous Wetland”, and “Moss and lichen”) or are not applicable (“Water”) are left out.

**Table 6:** Height error statistics per land cover class and all land covers combined for all corrected-DSMs and their sources as compared to local airborne lidar DTMs.

Land cover	n	bias [m]	MAE [m]	MAD [m]	RMSE [m]	<1 m [%]	<2 m [%]	<5 m [%]	DEM
Herbaceous wetland (11 %)	8549286	−0.03	1.35	1.02	1.87	49	79	98	NASADEM
	8264359	0.25	0.60	0.37	0.93	83	97	**100**	CopernicusDEM
	8545311	1.35	1.56	0.55	1.84	27	77	99	MERIT
	8547405	0.74	1.15	0.74	1.44	52	84	**100**	CoastalDEM
	8264359	0.26	0.56	0.34	0.82	84	98	**100**	FABDEM
	8263261	**0.00**	0.43	0.30	0.60	90	99	**100**	DiluviumDEM
	8264359	0.03	**0.36**	**0.28**	**0.49**	**95**	**100**	**100**	DeltaDTM
Grassland (32 %)	23372058	0.27	1.42	0.99	2.20	51	79	97	NASADEM
	23433669	0.34	0.77	0.39	1.60	78	93	99	CopernicusDEM
	23360478	1.11	1.40	0.65	1.76	40	79	99	MERIT
	23347032	−0.27	0.94	0.62	1.29	65	89	**100**	CoastalDEM
	23433669	0.19	0.64	0.36	1.14	80	96	99	FABDEM
	23431085	**−0.07**	0.58	0.33	0.97	83	96	**100**	DiluviumDEM
	23433669	**0.07**	**0.42**	**0.27**	**0.69**	**92**	**98**	**100**	DeltaDTM
Cropland (16 %)	11876998	−0.32	0.99	0.74	1.34	62	89	**100**	NASADEM
	11883267	−0.19	0.43	0.27	0.73	92	99	**100**	CopernicusDEM
	11876125	0.78	1.02	0.51	1.21	52	93	**100**	MERIT
	11875517	−0.47	0.75	0.47	1.00	73	95	**100**	CoastalDEM
	11883267	−0.18	0.38	0.24	0.63	95	**100**	**100**	FABDEM
	11883190	−0.25	0.55	0.28	1.02	87	95	99	DiluviumDEM
	11883267	**−0.07**	**0.32**	**0.23**	**0.44**	**97**	**100**	**100**	DeltaDTM
Built-up (8 %)	5909752	1.36	2.02	1.29	2.72	33	60	94	NASADEM
	5900020	1.59	1.73	0.87	2.41	39	69	96	CopernicusDEM
	5906062	2.56	2.68	1.13	3.21	16	40	90	MERIT
	5901961	−0.23	0.95	0.70	1.32	64	89	99	CoastalDEM
	5900020	0.22	0.69	0.47	1.04	79	95	**100**	FABDEM
	5899807	**−0.02**	0.80	0.51	1.26	75	92	99	DiluviumDEM
	5900020	−0.24	**0.57**	**0.37**	**0.91**	**86**	**96**	**100**	DeltaDTM
Tree cover (25 %)	18649418	3.83	4.26	2.44	5.94	21	39	69	NASADEM
	18617509	5.16	5.25	2.56	7.30	18	33	62	CopernicusDEM
	18634482	1.35	1.92	1.13	2.47	31	62	95	MERIT
	18620149	0.33	1.33	1.02	1.79	49	77	99	CoastalDEM
	18617509	1.81	2.14	1.16	3.29	42	65	90	FABDEM
	18616621	0.06	1.22	0.77	1.88	60	83	97	DiluviumDEM
	18617509	**0.04**	**0.56**	**0.36**	**0.95**	**87**	**96**	**100**	DeltaDTM
Mangroves (5 %)	4074981	3.37	3.79	2.22	5.21	22	40	72	NASADEM
	4019752	4.02	4.07	2.33	5.81	28	41	68	CopernicusDEM
	4054032	2.43	2.52	0.83	3.20	15	52	90	MERIT
	4038701	1.02	1.15	0.51	1.45	48	90	99	CoastalDEM
	4019752	1.84	1.89	0.96	2.60	42	63	94	FABDEM
	4019752	0.44	0.84	0.43	1.47	75	90	98	DiluviumDEM
	4019752	**0.17**	**0.48**	**0.31**	**0.73**	**89**	**97**	**100**	DeltaDTM
Overall	74875606	1.33	2.28	1.35	3.82	41	66	89	NASADEM
	73684832	1.78	2.10	0.74	4.11	59	74	87	CopernicusDEM
	74852660	1.34	1.68	0.78	2.18	35	71	97	MERIT
	74846548	0.05	1.06	0.79	1.44	59	86	99	CoastalDEM
	73148539	0.65	1.05	0.51	1.96	71	87	97	FABDEM
	73143642	0.02	0.72	0.40	1.22	79	93	99	DiluviumDEM
	73148539	**0.01**	**0.45**	**0.30**	**0.74**	**91**	**98**	**100**	DeltaDTM

**Bold** is best value(s) in each metric and class. Land cover is based on ESA WorldCover 2021 and classes with less than 1% of sampled data (“Water”, “Snow and Ice”, “Bare/sparse vegetation”, “Shrubland”, and “Moss and lichen”) are ignored. < m is within m of reference. n is the number of comparisons (pixels) and can differ per dataset due to the different watermasks in use.

DeltaDTM performs best for all land cover classes combined, with a bias of 0.01 m, a MAE of 0.45 m, and a RMSE of 0.74 m. 91% of DeltaDTM is within 1 m of the reference surface, 98% within 2 m and 100% within 5 m. The next best DEM is DiluviumDEM, followed by FABDEM–although closely matched by CoastalDEM, but not for the percentage within 1 m. DiluviumDEM has a bias of 0.02 m, a MAE of 0.72 m and a RMSE of 1.22 m, with 79% of values within 1 m. FABDEM has a bias of 0.65 m, a MAE of 1.05 m and a RMSE of 1.96 m, with 71% of values within 1 m. CoastalDEM, NASADEM, CopernicusDEM, and the MERIT DEM (described in Table 1) have lower accuracies (Table 6).

Comparison of validation scores over individual reference areas (Table 7) confirms the global validity of DeltaDTM across regions and climate zones. The overall MAE values of 9 of the 10 areas range from 0.350.59 m with only one apparent outlier (Marshall Islands) at 0.98 m. In all areas, the MAE of either the “Urban”, “Trees”, or “Mangroves” land cover is highest, and in nearly all the MAE of “Cropland” is the lowest.

**Table 7:** Height error statistics per land cover class and all land covers combined as per Table 6 split out for each validation area from Table 5.

Dataset	Land cover	n	bias [m]	MAE [m]	MAD [m]	RMSE [m]	<1 m [%]	<2 m [%]	<5 m [%]
Australia	Wetland	525357	−0.12	0.25	0.19	0.33	100	100	100
	Grassland	1195841	−0.20	0.36	0.23	0.49	97	100	100
	Cropland	21424	−0.15	0.30	0.23	0.39	99	100	100
	Urban	8141	0.01	0.94	0.72	1.25	62	90	100
	Trees	1628773	−0.08	0.78	0.41	1.27	76	90	99
	Mangroves	483255	0.74	0.96	0.66	1.35	64	85	100
	Overall	3952547	−0.02	0.59	0.31	0.99	85	94	100
Florida	Wetland	2213104	0.01	0.33	0.24	0.48	96	99	100
	Grassland	1933126	0.25	0.51	0.34	0.72	88	98	100
	Cropland	228976	0.15	0.28	0.17	0.46	95	99	100
	Urban	2556095	−0.07	0.49	0.34	0.73	89	98	100
	Trees	3750084	0.18	0.40	0.27	0.58	94	99	100
	Mangroves	2472533	0.19	0.35	0.25	0.49	95	100	100
	Overall	13208385	0.12	0.41	0.28	0.61	93	99	100
Indonesia	Wetland	199555	0.04	0.40	0.31	0.50	95	100	100
	Grassland	1679234	0.21	0.59	0.48	0.79	84	99	100
	Cropland	566366	−0.11	0.49	0.37	0.63	91	99	100
	Urban	28691	−0.01	0.44	0.36	0.60	92	100	100
	Trees	4684674	0.03	0.48	0.39	0.64	90	99	100
	Mangroves	223136	−0.07	0.51	0.37	0.67	89	99	100
	Overall	8198017	0.04	0.50	0.42	0.67	89	99	100
Marshall islands	Wetland	6	0.69	0.79	0.53	0.90	67	100	100
	Grassland	2842	0.47	0.89	0.59	1.11	64	93	100
	Urban	2460	0.28	0.99	0.48	1.22	59	89	100
	Trees	3481	0.95	1.06	0.52	1.26	51	92	100
	Overall	8939	0.61	0.98	0.52	1.20	57	91	100
Mexico	Wetland	4760852	0.07	0.39	0.31	0.50	95	100	100
	Grassland	4995129	0.42	0.57	0.34	0.80	85	98	100
	Cropland	175399	0.51	0.59	0.36	0.75	82	99	100
	Urban	193057	−0.09	0.67	0.48	0.95	80	95	100
	Trees	2425547	0.38	0.68	0.41	1.01	81	95	100
	Mangroves	839893	−0.17	0.56	0.40	0.82	85	97	100
	Overall	13415760	0.25	0.53	0.36	0.76	88	98	100
Netherlands	Wetland	385798	−0.11	0.29	0.20	0.44	97	99	100
	Grassland	11913519	−0.06	0.31	0.19	0.60	96	98	100
	Cropland	6399660	0.06	0.22	0.15	0.33	99	100	100
	Urban	2763729	−0.39	0.62	0.36	1.03	84	95	99
	Trees	3983525	−0.14	0.52	0.31	0.88	88	96	100
	Overall	25476046	−0.08	0.35	0.21	0.66	94	98	100
United Kingdom	Wetland	36780	−0.92	0.94	0.26	1.04	60	98	100
	Grassland	784332	−0.52	0.61	0.24	0.82	89	97	100
	Cropland	2777995	−0.52	0.53	0.17	0.60	97	100	100
	Urban	145781	−0.70	0.80	0.35	1.17	76	94	99
	Trees	261257	−0.40	0.75	0.37	1.14	79	94	99
	Overall	4009925	−0.52	0.58	0.19	0.72	93	99	100

Land cover is based on ESA WorldCover 2021 and classes with less than 1% of sampled data (“Water”, “Snow and Ice”, “Bare/sparse vegetation”, “Shrubland”, and “Moss and lichen”) are ignored. < m is within m of reference. n is the number of comparisons (pixels). Trees is “Tree cover”, while Wetland is “Herbaceous wetland”. The full table–including datasets from Poland and Latvia–is included as Supplementary Table 1.

Each DEM has its own strengths and performs differently per land cover class. For example, FABDEM has been optimized for urban areas and has a similar performance for “Built-up” as DeltaDTM, with a MAE of 0.69 m and 0.61 m respectively.

In areas with no vegetation or buildings, like “Wetland” or “Cropland”, an uncorrected DSM such as CopernicusDEM performs similar to corrected-DSMs. CopernicusDEM has a MAE of 0.43 m for “Cropland”, whereas FABDEM and DeltaDTM have a MAE of 0.38 m and 0.32 m, respectively.

As expected, the errors for “Tree cover” are greatest, with 87% of DeltaDTM elevations within 1 m, one of its lowest values overall. DiluviumDEM is next, with 60% within 1 m, followed by CoastalDEM at 49%, and FABDEM at 42%. Notably, FABDEM has a lower accuracy in “Tree cover” at 2.14 m MAE than MERIT at 1.92 m MAE and CoastalDEM at 1.33 m MAE. DeltaDTM has a 0.56 m MAE in “Tree cover” (Table 6).

To understand the impact of our methodological choices, we have provided the impact on the accuracy (MAE) and the percentage of cells affected by each processing step in Table 8. Overall, the classification step in filtering non-ground cells is the most important, improving the MAE by 1.58 m. This is followed by the *burn* step in the same filtering non-ground cells category when measured individually, as not using ICESat-2 and GEDI values (only accounting for 4% of cells) for the subsequent classification would worsen the MAE by 0.62 m. The preprocessing steps to remove outliers are of little quantitive impact overall (not improving the MAE, affecting 1% of cells) and mainly serve to prevent visual artefacts.

**Table 8:** The impact of each processing step of DeltaDTM on the overall MAE and the number of cells affected when compared to reference areas.

Measured	Step	Clarification of disabled step	MAE [m]	Δ MAE [m]	cells [%]
Individually	None	No steps disabled (equals DeltaDTM)	0.45		
	Low filter	Removing low outliers that could impact further processing	0.45	0.00	1
	Bias correction	Correction of vertical bias present in CopernicusDEM	0.51	−0.06	100
	Burn	Use ICESat-2 and GEDI elevations where available	1.07	−0.62	4
	Classify + interpolation	Remove non-terrain cells and fill the resulting voids	1.72	−1.27	50
Cumulatively	None	None (equals CopernicusDEM)	2.10		
	Low filter	Low Filter	2.10	0.00	1
	Bias correction	Low Filter + Bias correction	2.04	−0.06	100
	Burn	Low Filter + Bias correction + Burn	2.03	−0.01	100
	Classify + interpolation	Low Filter + Bias correction + Burn + Classify + interpolation (equals DeltaDTM)	0.45	−1.58	100

Measured both individually by disabling each processing step separately in DeltaDTM, and cumulatively by applying processing steps sequentially on CopernicusDEM. Δ MAE is the difference in MAE compared to DeltaDTM for the steps measured individually, and the difference with the previous step for cumulatively measured steps. The classification and interpolation step has by far the most impact on the MAE.

### Qualitative visual validation

Apart from the quantitative validation, we also perform a qualitative visual validation of the DeltaDTM dataset. Such a validation is important to ensure that the dataset is free from artefacts and is realistic, since relying on a limited set of metrics can be misleading^37,38^.

Figures 8, 9 show the reference dataset, a land cover map from ESA Worldcover, and the corrected-DSMs MERIT, CoastalDEM, FABDEM, DiluviumDEM and DeltaDTM and their differences with the reference for reference areas. These figures also include a hillshade visualisation of the DEM to efficiently assess the ability of the DEMs to represent the landscape^37^. We use Perceptually Shaded Slope Maps (PSSM)^39^, which gives much more contrast in slopes than default hillshade visualisations, which is essential in areas with little to no relief as present in the LECZ. The remainder of the figures for all validation areas can be found as Supplementary Figures 1–17. The legend for the ESA Worldcover map is given in Supplementary Figure 18.

**Fig. 8:**
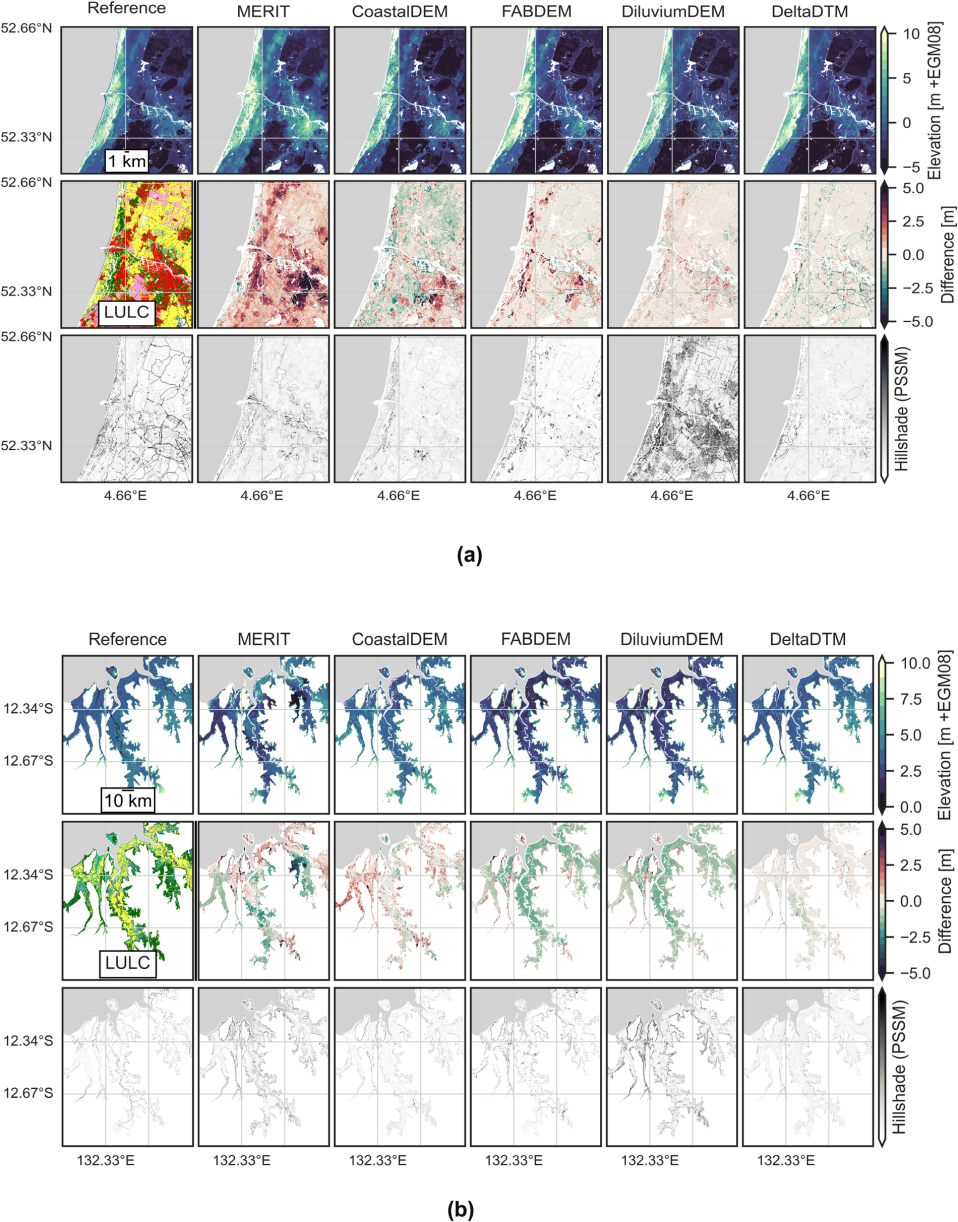
A comparison of corrected-DSMs in validation areas with (**a**) embankments in North-Holland, the Netherlands and (**b**) large vertical bias differences in the original DSMs used as input in the Northern Territories, Australia. The top row shows DEMs, while the center row shows the difference with the reference elevation in the top left. The ESA WorldCover map is given for context in the center left. The bottom row shows the hillshades for all DEMs. (**a**) Note how the embankments (highways, such as the peripheral road) around Amsterdam are removed in all corrected-DSMs and DeltaDTM, and show up as negative (green) in the difference with the reference map. (**b**) FABDEM and DiluviumDEM (both based on CopernicusDEM) are two meters lower than the reference, while the bias correction in DeltaDTM (also using CopernicusDEM) mostly negates this bias.

**Fig. 9:**
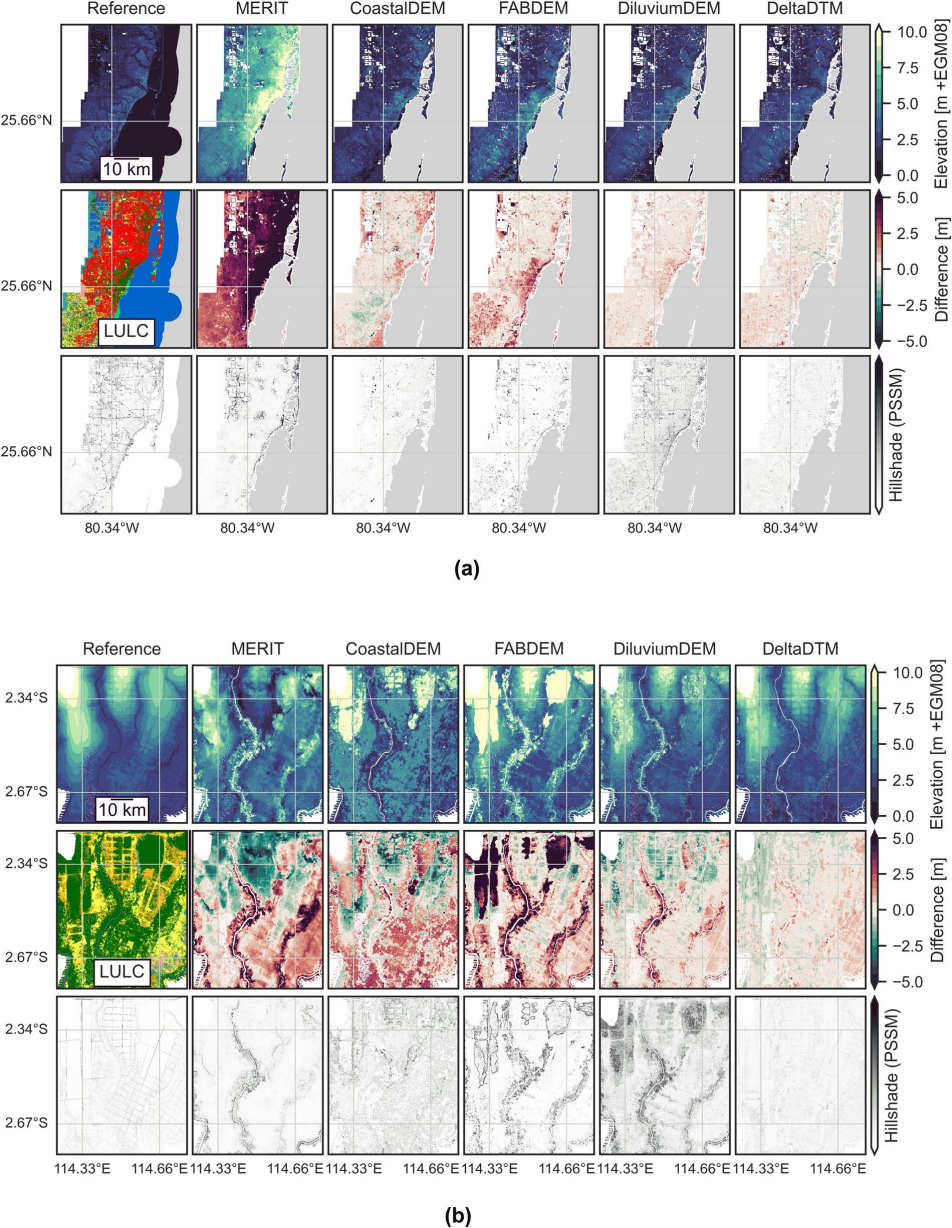
A comparison of corrected-DSMs in the validation areas with (**a**) most Built-up land cover in Florida, USA and (**b**) the one with most “Tree cover” in Kalimantan, Indonesia. The top row shows DEMs, while the center row shows the difference with the reference elevation in the top left. The ESA WorldCover map is given for context in the center left. The bottom row shows the hillshades for all DEMs. (**a**) Urban coastline of Miami, on average 2 m + MSL. (**b**) All corrected-DSMs have difficulty with the dense vegetation. The hatched patterns in the DTM and to some extent in the corrected-DSMs are drainage canals in the peat domes.

In Florida, the United States (Fig. 9a), the area with the most built-up land cover, DeltaDTM performs best of the corrected-DSMs. MERIT is too high overall, but mostly so in the Built-up area. CoastalDEM tends to be more balanced, lower in the south, slightly higher along the coastline. FABDEM performs well in the urban area, but is higher than the reference for the vegetated parts of the coast. DiluviumDEM is on par with DeltaDTM, but slightly higher, and has a higher average slope.

In Kalimantan, Indonesia (Fig. 9b), the reference area with most “Tree cover”, all datasets have lower accuracies. Clearly, extensive and dense forest in the tropics is hard to correct for. MERIT tends to overcorrect the forest on the peat dome, making it too low, while missing the vegetation along the canals. CoastalDEM shows a similar range of errors, without a clear pattern. FABDEM has the largest positive errors, not always correcting larger patches of forests or the vegetation along the canals. DiluviumDEM removes the larger patches of forest, but overcorrects, becoming too low overall. DeltaDTM is closest to the reference, having the smallest errors overall but still misses smaller patches of forest. We attribute these errors in small patches to a misclassification of the (“open” versus “closed”) land cover class–like cropland instead of tree cover–resulting in different (less strict) filter settings.

In other validation areas (Fig. 8), we find two patterns of interest. First, in the Netherlands (Fig. 8a), the embankments of major highways tend to be removed in all corrected-DSMs. This effect is especially pronounced in DeltaDTM, as it is the only major source of errors. Notably, as demonstrated in the hillshades, DiluviumDEM creates very rough terrain where corrections are applied. Second, in Australia (Fig. 8b), we find that CopernicusDEM–used as input for both FABDEM, DiluviumDEM, and DeltaDTM–is two metres lower than the reference. FABDEM and DiluviumDEM are not able to correct this bias and are also two metres too low, whereas the bias correction step in DeltaDTM can negate this error in the source dataset.

When comparing hillshades across the validation areas, all corrected-DSMs smooth landscape details–such as infrastructure and canals–present in the reference DTM. Moreover, the hillshades indicate steep slopes caused by pits and patches of forest in the corrected-DSMs. In the validation area of Kalimantan, Indonesia (Fig. 9b)–the smoothest terrain overall–the hillshades show artefacts in the processing of CoastalDEM and DiluviumDEM. CoastalDEM and DiluviumDEM have square patches of pixels that differ from one another, whereas DiluviumDEM also has large differences between corrected-pixels, resulting in a high overall slope.

**Fig. 10:**
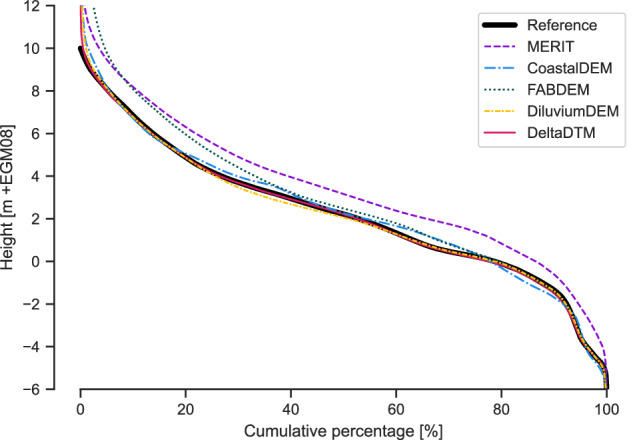
Hypsography (cumulative distribution function of height) for all validation areas combined. For the reference line, 60% (20%–80%) of all values are between 05 m. All corrected-DSMs follow the trend of the reference, but are generally higher, especially for values above 10 m. MERIT is consistently higher than the reference, whereas FABDEM is mostly higher for values above 4 m. Both CoastalDEM and DeltaDTM follow the reference closely, but CoastalDEM oscillates more, and is the only corrected-DSM that is visibly lower than the reference on occasion.

When we compare all elevation values as a cumulative distribution (a hypsography) as in Fig. 10, we see that all corrected-DSMs follow the trend of the reference, but are generally higher, especially for values above 10 m + MSL. MERIT is consistently higher than the reference, whereas FABDEM is mostly higher for values above 4 m + MSL. Both CoastalDEM, DiluviumDEM and DeltaDTM follow the reference closely, but CoastalDEM and DiluviumDEM oscillate more, and are the only corrected-DSMs that are visibly lower (containing fewer values) than the reference.

### Cross-validation against ICESat-2

The airborne lidar reference datasets used to validate DeltaDTM (Table 5 and Fig. 7) are geographically limited. Indeed, we found only two datasets for the whole Global South, while most of the tree land cover–and thus the largest biases in DSMs–occur there. We thus choose to validate against ICESat-2 as it has global coverage.

However, because DeltaDTM directly incorporates ICESat-2 measurements, we cannot directly use ICESat-2 for validation. Instead, we cross-validate by using a modified version of DeltaDTM, generated with only 66 of ICESat-2 data and use the remainder 33 for validation. Specifically, we leave out track two (gt2l, gt2r) of all ICESat-2 data, which is the beam pair (one strong, one weak beam) in the middle. Leaving out whole granules will cause an imbalance in the global coverage, while leaving out other beam(pair)s causes imbalances in the beam power distribution and could allow for validation only 90 m away from training data. Note that these results are thus for a modified version of DeltaDTM, the actual version has better performance.

The datasets involved are not independent, and this cross-validation could in theory overestimate the accuracy of DeltaDTM by ignoring any biases in ICESat-2. However, there is no other global validation dataset, and we are confident in the vertical accuracy of ICESat-2. For example, we find a MAE of 0.44 m when we compare ICESat-2 measurements to the airborne lidar dataset of the Netherlands and Liu *et al*.^32^ found a MAE of 0.91 m when they compared ICESat-2 to airborne lidar datasets across the USA. GEDI is not used for cross-validation, as it is less accurate (MAE of 1.80 m^32^) and does not cover the higher latitudes.

We reuse the metrics from the previous validation, also separated per land cover class, for DeltaDTM (Table 9). In comparison with global ICESat-2 measurements, DeltaDTM has a mean error of 0.13 m, a MAE of 0.75 m, a MAD of 0.32 m and a RMSE of 3.27 m. 83% of all samples are within 1 m of ICESat-2, and 94% and 99% within 2 m and 5 m respectively. These measurements–apart from the RMSE–are similar to the validation statistics in Table 6.

**Table 9:** Height error statistics for cross-validation with ICESat-2 per land cover class and all land covers combined.

Land cover	n	bias [m]	MAE [m]	MAD [m]	RMSE [m]	<1 m [%]	<2 m [%]	<5 m [%]
Bare/sparse vegetation (14%)	25507870	0.18	0.65	0.23	3.70	90	96	99
Herbaceous Wetland (27%)	51086453	−0.26	0.49	0.29	0.79	87	97	100
Grassland (25%)	47376473	0.08	0.46	0.26	1.21	90	97	100
Cropland (14%)	25930835	0.09	0.37	0.23	0.67	93	99	100
Tree cover (13%)	24556841	0.62	1.21	0.53	3.16	69	85	96
Shrubland (2%)	3549083	−0.07	0.54	0.32	1.01	86	96	100
Snow and ice (1%)	943697	12.16	12.90	3.28	25.98	28	41	60
Mangroves (3%)	5533679	0.40	0.72	0.36	1.52	81	93	98
Moss and lichen (1%)	2361855	0.08	0.67	0.34	2.32	85	94	99
Overall	186846786	0.13	0.75	0.32	3.27	83	94	99

<m means within m to reference. Note how the “Snow and ice” land cover class has the worst performance. Overall, the performance is similar to the validation with local DTMs, indicating that DeltaDTM does perform well globally.

**Fig. 11:**
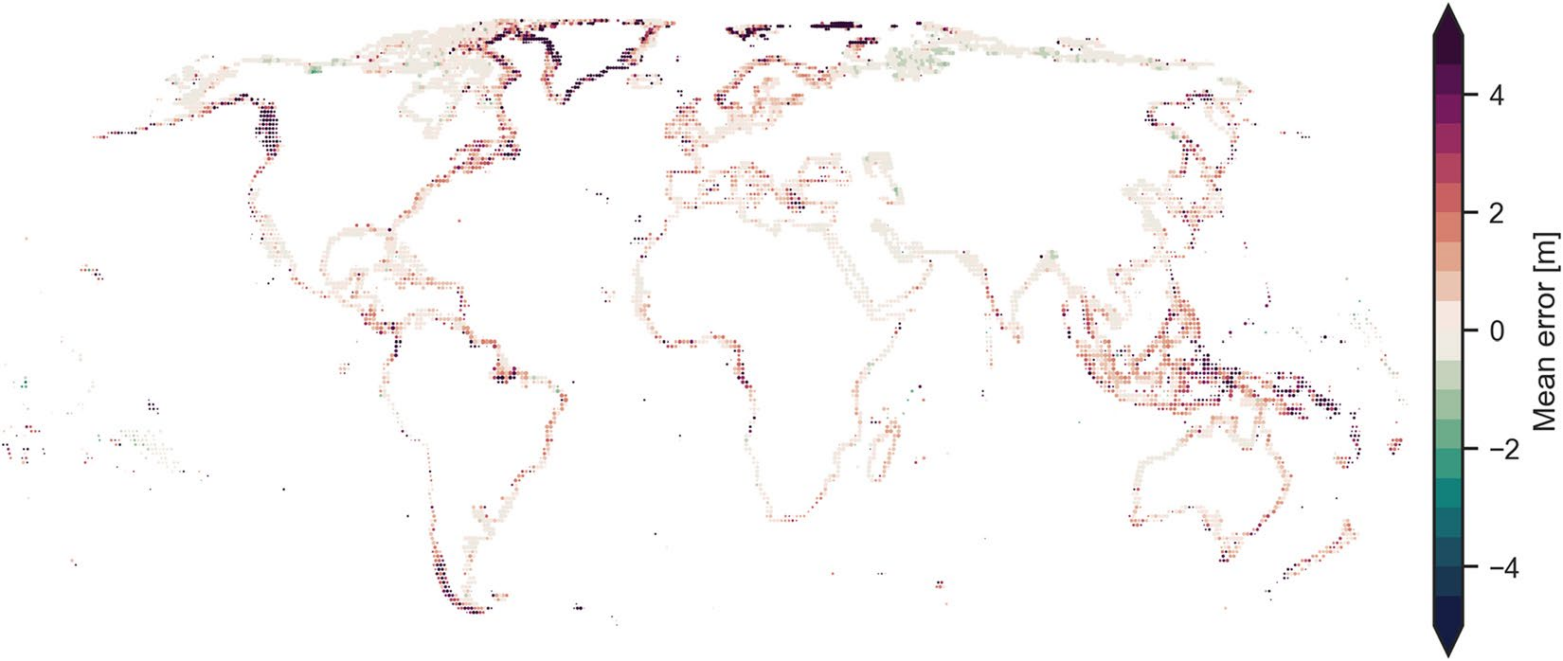
Global mean error of DeltaDTM per tile when cross-validated against ICESat-2 as points. Each tile is logarithmically sized to the number of samples, so steeper coasts (fewer samples below 10 m) and islands are represented by smaller points. Note how the overall error is close to zero, whereas larger errors occur especially in the higher latitudes with ice cover, such as Greenland, Canada, and Argentina and steep mountainous coasts. The white boundaries between tiles are for visualisation only, in reality tiles are connected.

The high overall RMSE of 3.27 m (within 1 m for the validation with local DTMs) is explained by a high number of outliers, that are less prominent in other statistical measures. These outliers occur either in DeltaDTM or in ICESat-2 data used for validation, and especially at steeper coasts, as seen in Fig. 11. Note also that the error of “Snow and ice” (MAE of 12.90 m) is substantially worse than that of any other land cover class (MAE typically within 1 m). We attribute this to the dynamic nature of the cryosphere–given that CopernicusDEM base dataset for DeltaDTM is roughly ten years old–and the interaction of the X-band radar of CopernicusDEM with ice. The “Tree cover” class has the second-largest errors (MAE of 1.21 m), which is the most difficult land cover class to correct for in all corrected-DSMs, as shown in the validation against reference datasets. We note that ICESat-2, used here as reference, also has decreased accuracy in closed land cover classes^32^, which further contributes to the error.

## Usage Notes

Like any global product, the DeltaDTM dataset contains outliers and artefacts. Because of the high accuracy of DeltaDTM, errors in resolving smaller features stand out for the first time. This is clearest in Fig. 8a, where DeltaDTM has removed the embankments of major highways. Indeed, whereas previous corrected-DSMs tend to overestimate the elevation due to the presence of forests and urban areas (errors of omission), DeltaDTM will tend to underestimate the elevation because it mistakenly removes these embankments (errors of commission). Furthermore–while better than other DEMs in correcting the bias due to vegetation–we still see the largest errors in the “Tree Cover” land cover class. We intend to improve these aspects of DeltaDTM in subsequent versions.

**Fig. 12:**
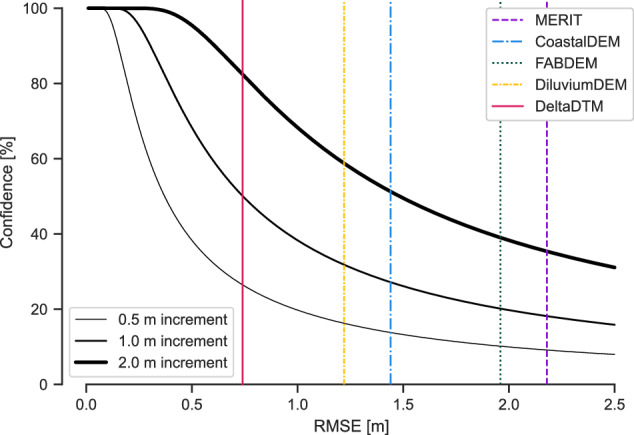
The confidence level associated with modelling SLR in increments of 0.52 m given the vertical uncertainty (RMSE) of a DEM^5^. The overall RMSE for all corrected-DEMs (Table 6) is given. DeltaDTM can be used to model SLR in increments of 1 m at 50% confidence.

It should be realized that given the overall RMSE of 0.74 m, DeltaDTM can be used to model SLR in increments of 1.48 m or higher at 68% confidence level^5^. For 1 m SLR increments, confidence level will be 50% (Fig. 12). DeltaDTM should not be used for areas in the arctics, given the large errors of CopernicusDEM there. Because of the cut-off above 10 m + MSL–we consider DeltaDTM potentially unfit for certain terrain analyses such as extracting drainage networks. Otherwise, the processing (Fig. 1) leaves no voids left other than nodata values for oceans, lakes, and rivers. These masked water bodies can be identified in the mask tiles provided with the dataset (with 0 being land, 1 ocean, 2 lakes, and 3 rivers), so that users can make the dataset continuous if needed.

DeltaDTM is licensed under CC BY 4.0 licence, which means that you are free to share and adapt the dataset, as long as you give appropriate credit (i.e. cite this paper). DeltaDTM is produced using Copernicus WorldDEM-30 © DLR e.V. 2010–2014 and © Airbus Defence and Space GmbH 2014–2018 provided under COPERNICUS by the European Union and ESA; all rights reserved.

While a virtual raster (DeltaDTM.vrt) with a single resolution linking all individual tiles is provided, note that individual DeltaDTM tiles have different sizes (and thus slightly different longitudinal resolutions) depending on the latitude, following the original CopernicusDEM (DGED^40^) tiling scheme. That is, to account for the curvature of the earth, the tiles are smaller in width at higher latitudes. From 0–50 degrees latitude, tiles have 3600 × 3600 pixels, while from 50–60 degrees latitude, tiles have 3600 × 2400 pixels, and become smaller from there on. All sizes are given in Table 10. The virtual raster cells can thus appear stretched when opened in a GIS environment. It is advised to use individual tiles when possible.

**Table 10:** Tile sizes for each DeltaDTM tile per latitude range.

Latitude	Tile width (columns)	Tile height (rows)
0° to 50°	3600	3600
50° to 60°	2400	3600
60° to 70°	1800	3600
70° to 80°	1200	3600
80° to 85°	720	3600
85° to 90°*	360	3600

Tiles narrow with increasing latitude, to keep the longitudinal resolution around 30 m. We follow the CopernicusDEM convention here, itself based on the DGED specification^40^. *No DeltaDTM tiles exist for this latitude range.

### Supplementary information

Supplymentary Figures 1-18

Supplymentary Table 1

## Data Availability

All code developed for this study is openly available as DeltaDTM.jl^41^ at https://zenodo.org/doi/10.5281/zenodo.10051451 under the GNU General Public License v3.0. The code is written in the Julia programming language^42^.
