# Supplementary material for: DeltaDTM: A global coastal digital terrain model
Source: Sci Data. 2024 Mar 6;11:273. doi: 10.1038/s41597-024-03091-9 (PMC10917791; doi:10.1038/s41597-024-03091-9)
Supplement: Supplementary file 1 — Supplymentary Figures 1-18 [file 41597_2024_3091_MOESM1_ESM.pdf]

Supplementary Figures

List of Supplymentary Figures

1 Marshall Islands, Majuro 2
2 Mexico, Tabasco 3
3 Mexico Tabasco 2 3
4 USA, Florida 4
5 USA, Florida 2 4
6 United Kingdom, The Fens 5
7 United Kingdom, The Fens 2 5
8 the Netherlands, Zeeland 6
9 the Netherlands, Flevoland 6
10 the Netherlands, Friesland 7
11 Poland, Gdansk 7
12 Poland, Gdansk 2 8
13 Latvia, Jūrmala 8
14 Indonesia, Sumatra 9
15 Indonesia, Kalimantan 9
16 Australia, Cocos Island 10
17 Australia, Darwin 10
18 Legend of ESA WorldCover 11

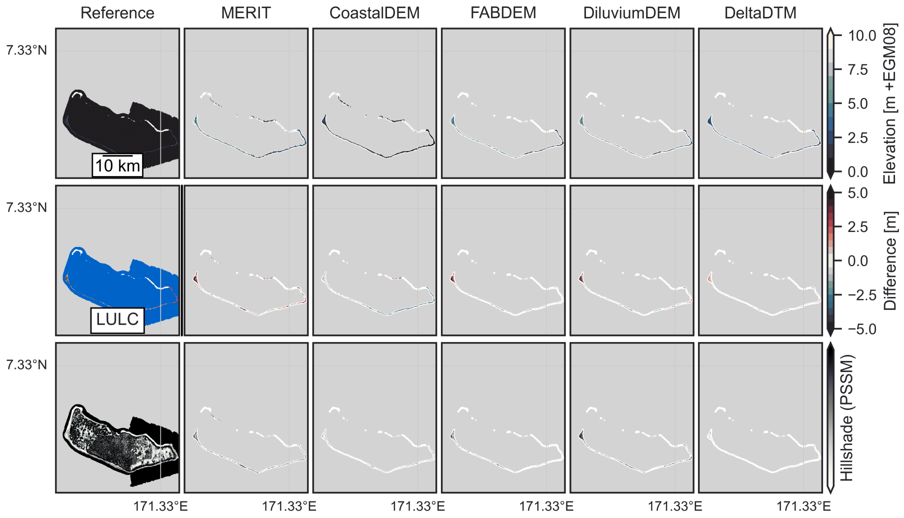

Supplementary Figure 1: Marshall Islands, Majuro

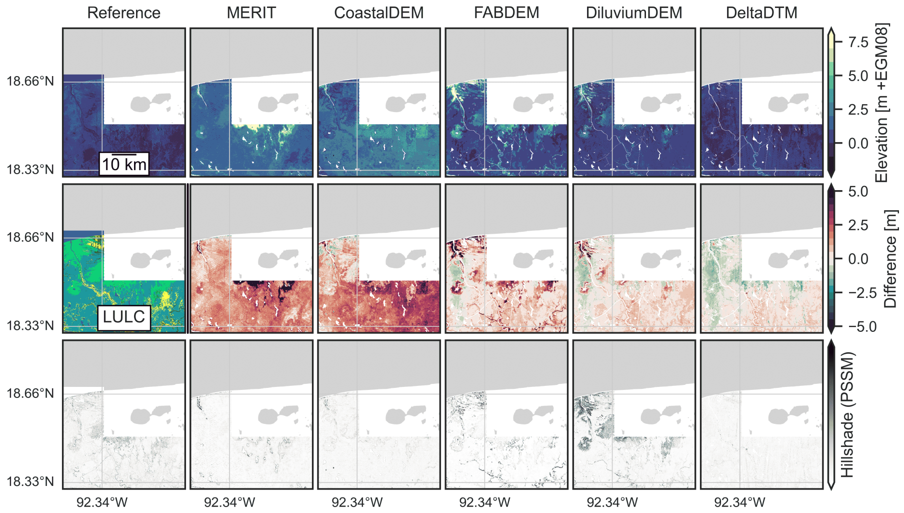

Supplementary Figure 2: Mexico, Tabasco

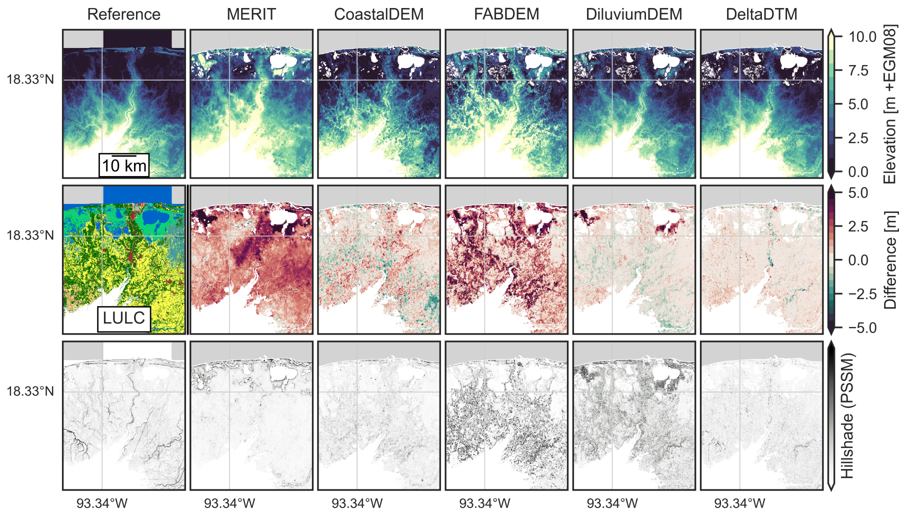

Supplementary Figure 3: Mexico Tabasco 2

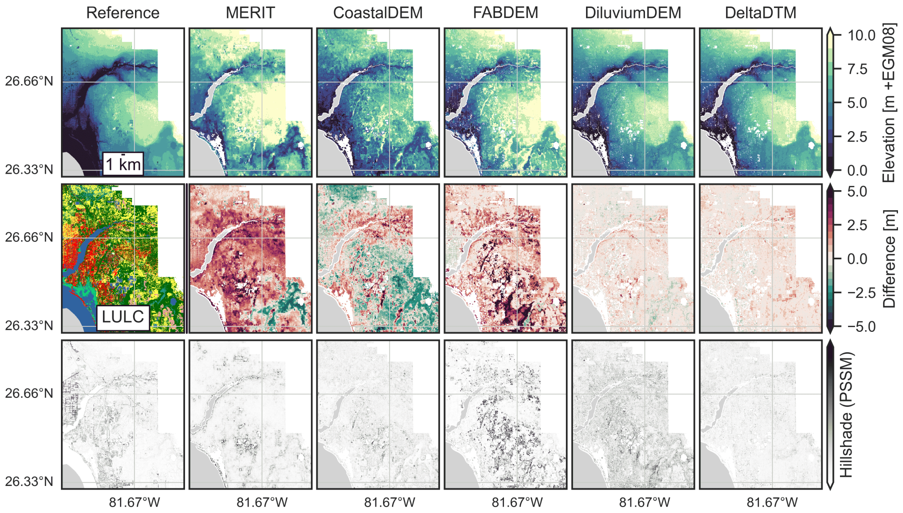

Supplementary Figure 4: USA, Florida

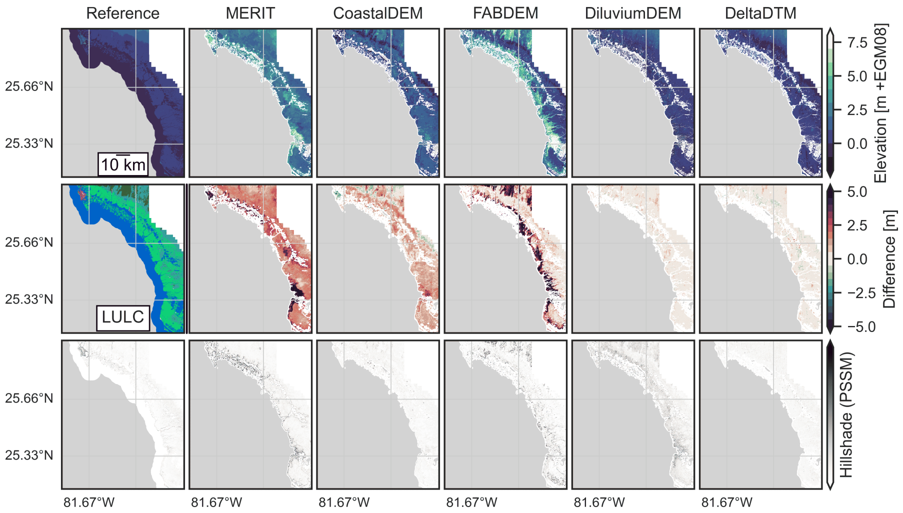

Supplementary Figure 5: USA, Florida 2

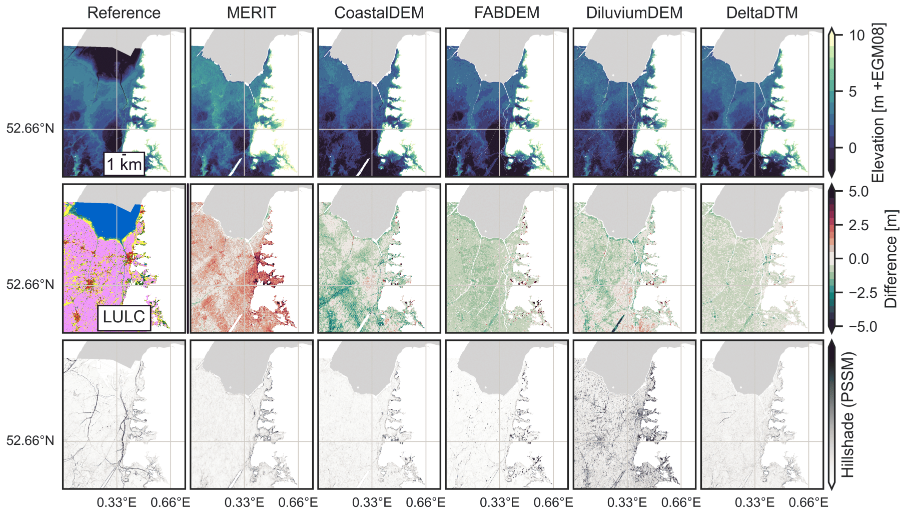

Supplementary Figure 6: United Kingdom, The Fens

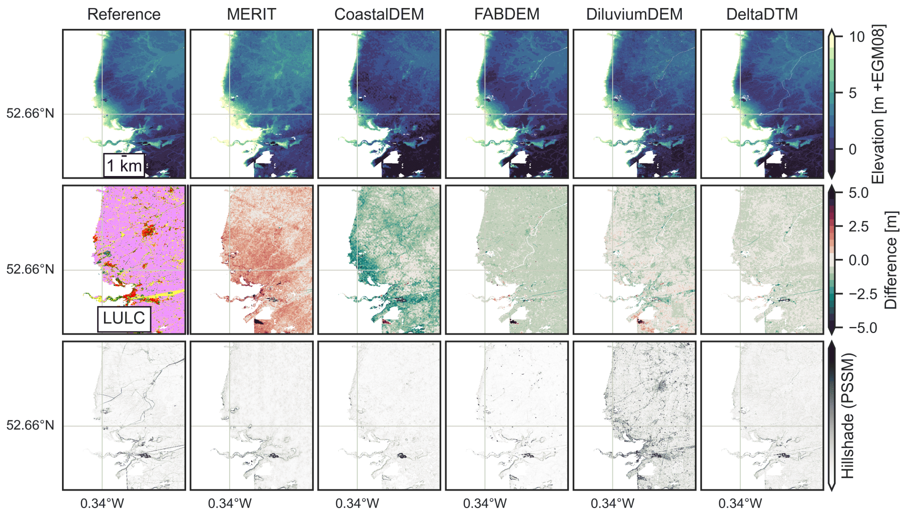

Supplementary Figure 7: United Kingdom, The Fens 2

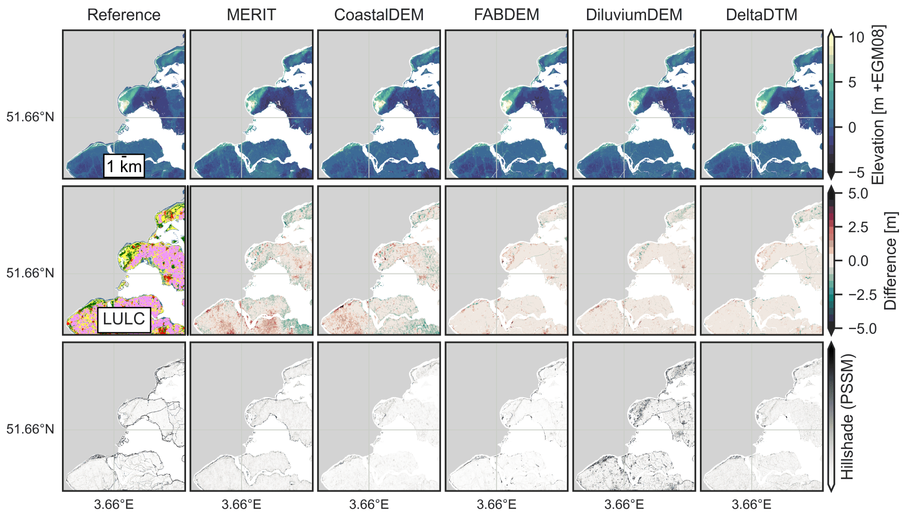

Supplementary Figure 8: the Netherlands, Zeeland

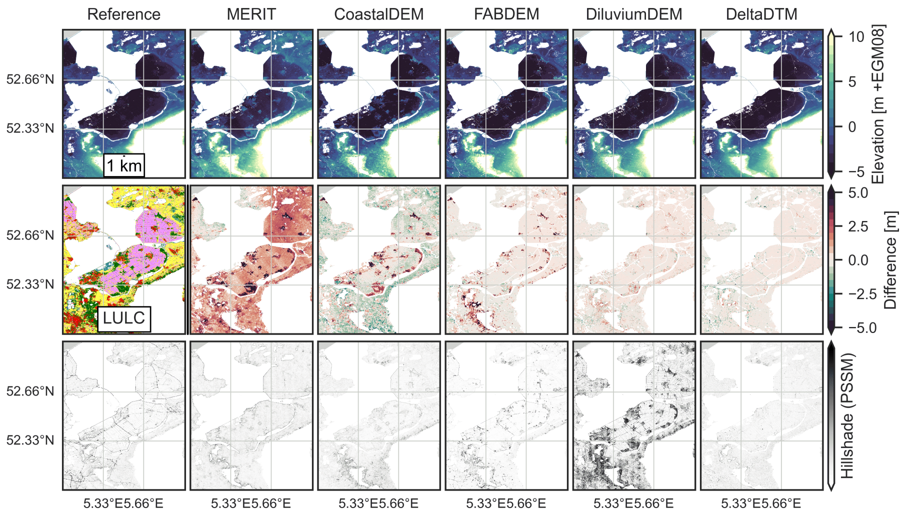

Supplementary Figure 9: the Netherlands, Flevoland

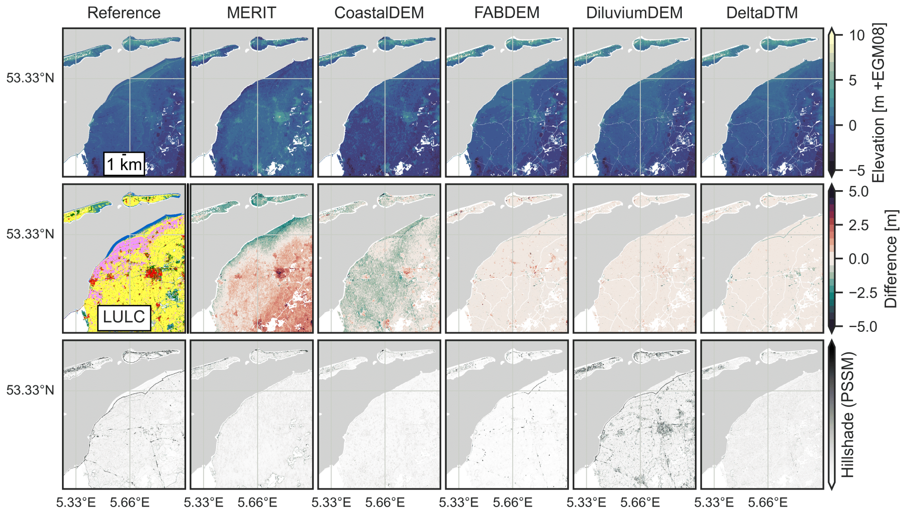

Supplementary Figure 10: the Netherlands, Friesland

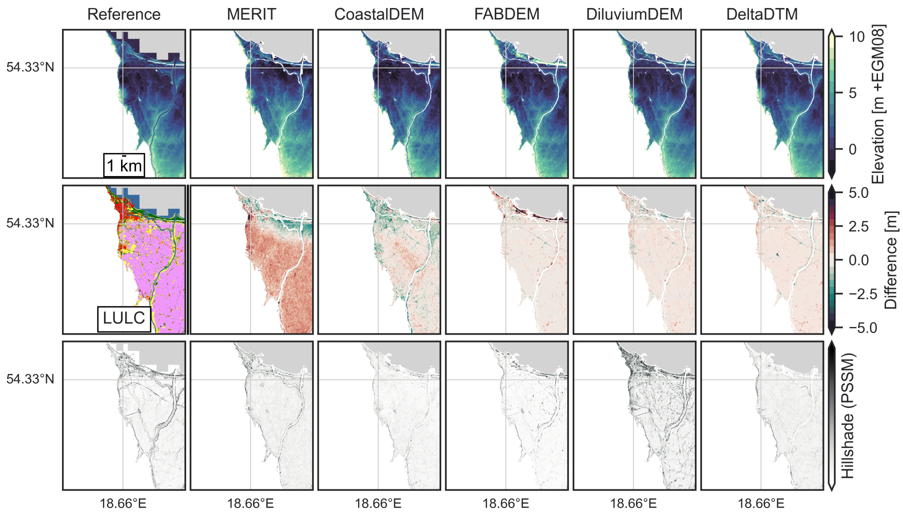

Supplementary Figure 11: Poland, Gdansk

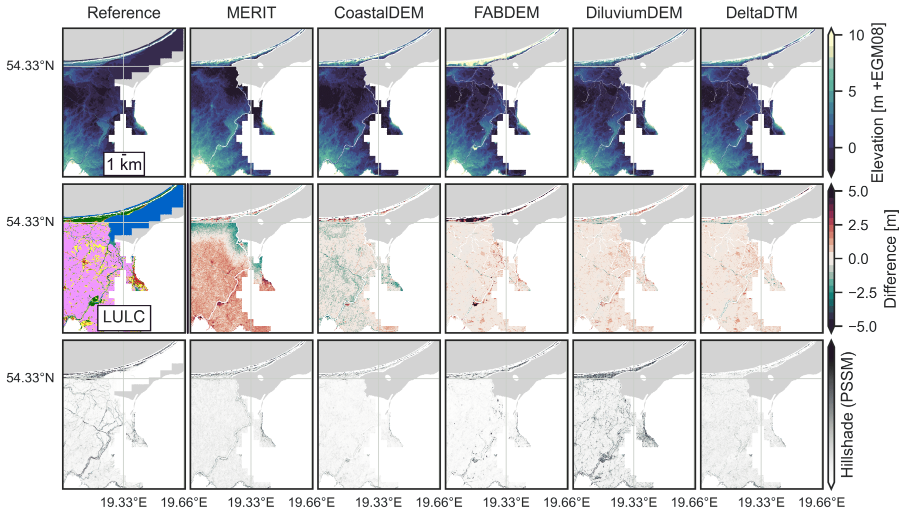

Supplementary Figure 12: Poland, Gdansk 2

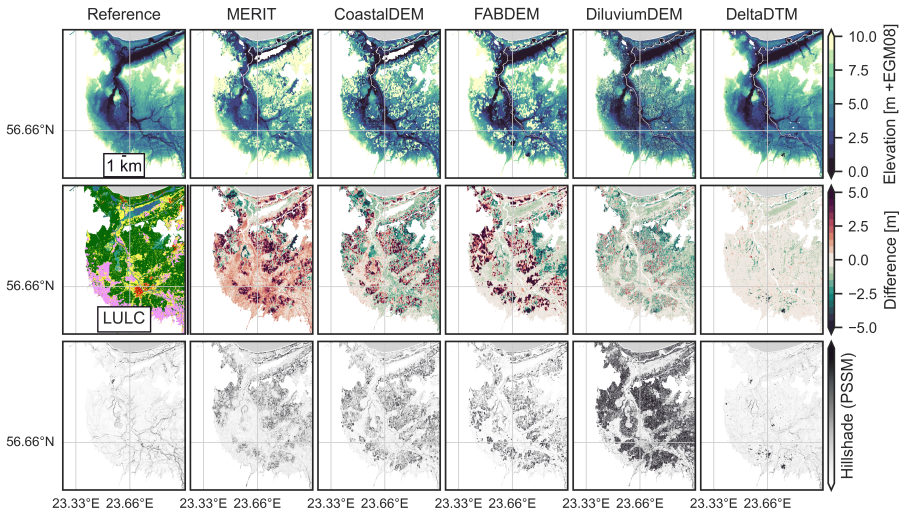

Supplementary Figure 13: Latvia, Jūrmala

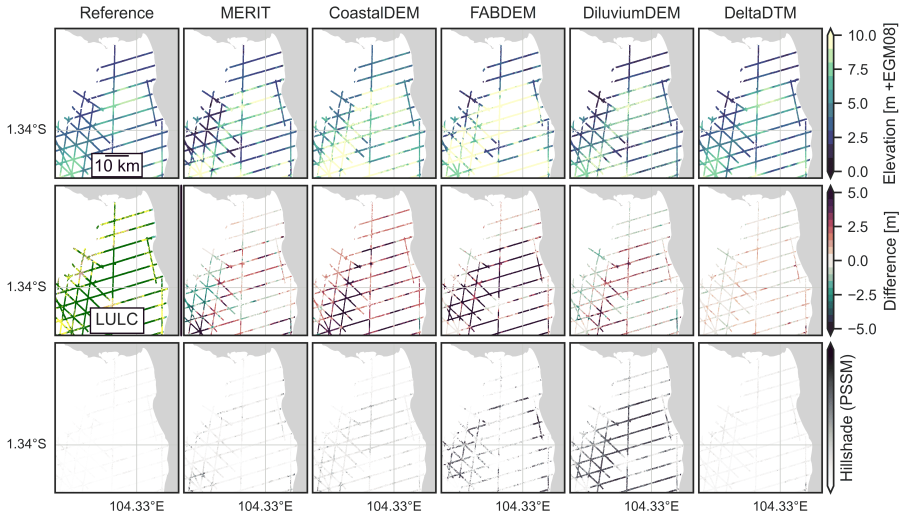

Supplementary Figure 14: Indonesia, Sumatra

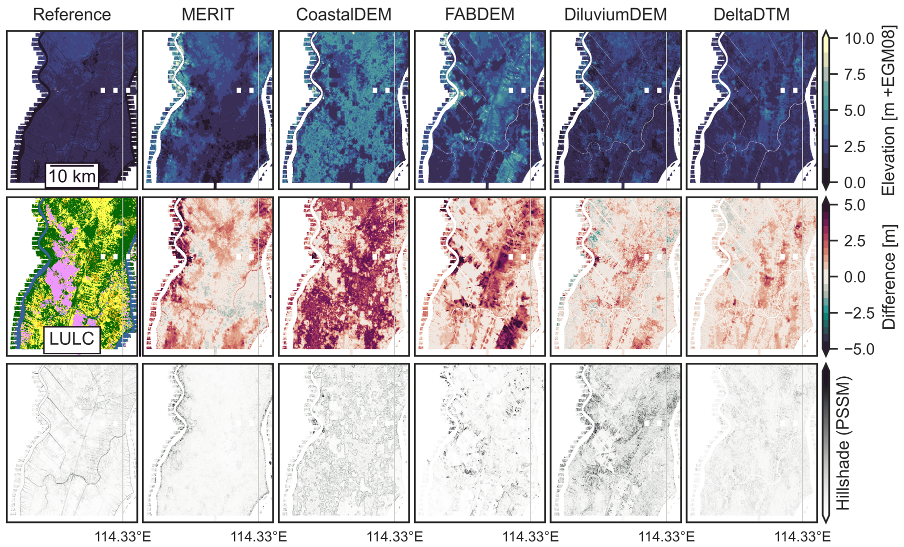

Supplementary Figure 15: Indonesia, Kalimantan

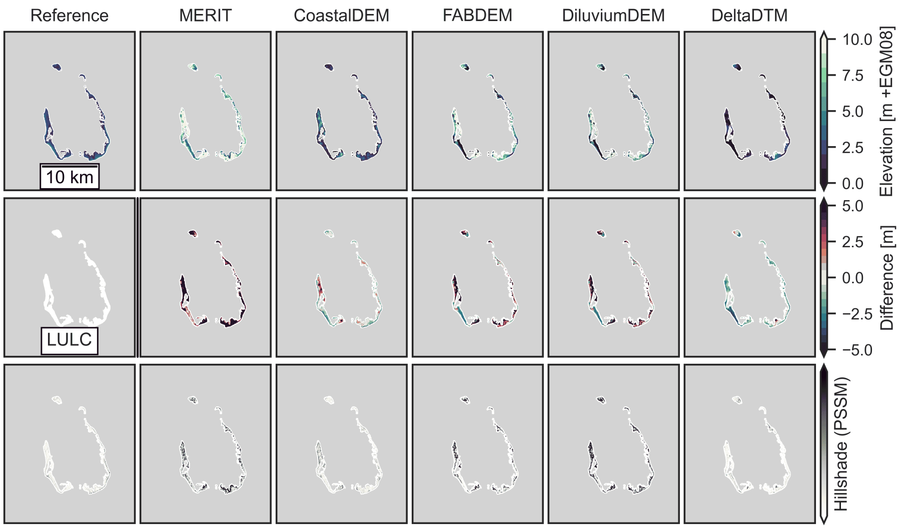

Supplementary Figure 16: Australia, Cocos Island

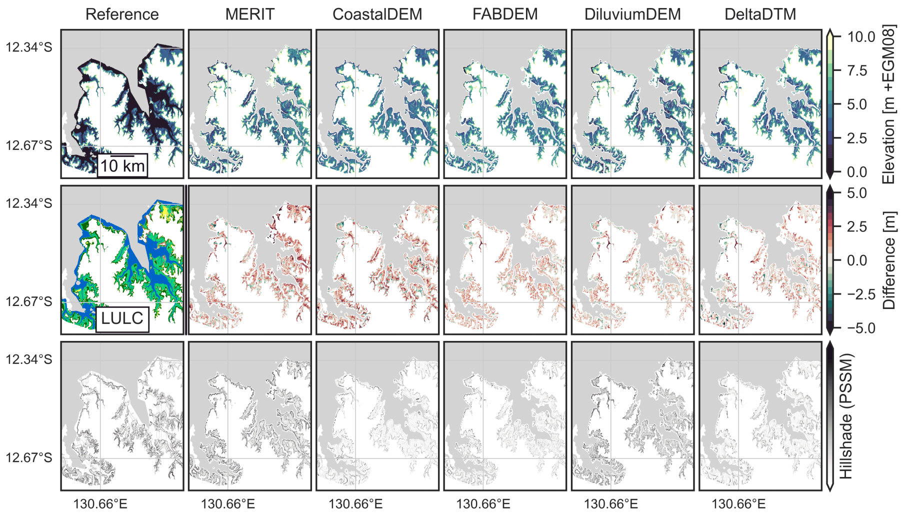

Supplementary Figure 17: Australia, Darwin

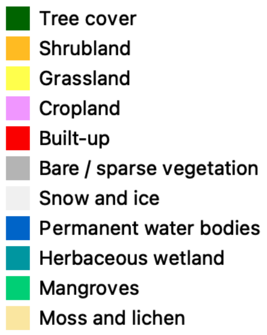

Supplementary Figure 18: Legend of ESA WorldCover
